# Mesenchymal Stem Cells for Neurological Disorders

**DOI:** 10.1002/advs.202002944

**Published:** 2021-02-24

**Authors:** Anna Andrzejewska, Sylwia Dabrowska, Barbara Lukomska, Miroslaw Janowski

**Affiliations:** ^1^ NeuroRepair Department Mossakowski Medical Research Centre PAS Warsaw 02‐106 Poland; ^2^ Center for Advanced Imaging Research Department of Diagnostic Radiology and Nuclear Medicine University of Maryland Marlene and Stewart Greenebaum Comprehensive Cancer Center University of Maryland Baltimore MD 21201‐1595 USA; ^3^ Tumor Immunology and Immunotherapy Program University of Maryland Marlene and Stewart Greenebaum Comprehensive Cancer Center University of Maryland Baltimore MD 21201‐1595 USA

**Keywords:** cell engineering, homing, mesenchymal stem cells, migration, neurological disorders, regeneration, transplantation

## Abstract

Neurological disorders are becoming a growing burden as society ages, and there is a compelling need to address this spiraling problem. Stem cell‐based regenerative medicine is becoming an increasingly attractive approach to designing therapies for such disorders. The unique characteristics of mesenchymal stem cells (MSCs) make them among the most sought after cell sources. Researchers have extensively studied the modulatory properties of MSCs and their engineering, labeling, and delivery methods to the brain. The first part of this review provides an overview of studies on the application of MSCs to various neurological diseases, including stroke, traumatic brain injury, spinal cord injury, multiple sclerosis, amyotrophic lateral sclerosis, Alzheimer's disease, Huntington's disease, Parkinson's disease, and other less frequently studied clinical entities. In the second part, stem cell delivery to the brain is focused. This fundamental but still understudied problem needs to be overcome to apply stem cells to brain diseases successfully. Here the value of cell engineering is also emphasized to facilitate MSC diapedesis, migration, and homing to brain areas affected by the disease to implement precision medicine paradigms into stem cell‐based therapies.

## Graphical abstract

Nowadays, an aging society struggles with a spectrum of incurable neurological disorders. Experimental therapies with mesenchymal stem cells (MSC) are evaluated in central nervous system pathologies with promising results. However, there are still unresolved issues with low MSCs delivery and engraftment rate. Several prevention strategies are developed for this issue, which the reader may get acquainted with herein.

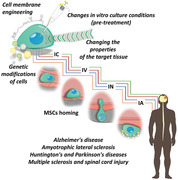

## 1 Introduction

**Figure 1:**
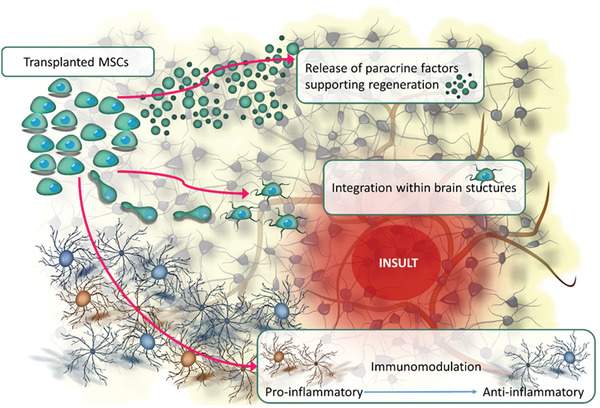
MSCs activities facilitating regeneration in neurological diseases.

Neurological impairments are usually irreversible due to limited regeneration in the central nervous system (CNS). The scope of treatment options for neurological diseases is restrained compared with other conditions. Recently, stem cell therapy has provided hope for many patients. Based on stem cells' regenerative capacity, transplantation therapies of various stem cells have been tested in basic research and preclinical studies, and some have shown great promise. At one time, neural stem cells (NSCs) seemed to be an optimal choice for therapeutic intervention in the central nervous system; however, to date, the majority of studies report trophic and immunomodulatory effects rather than neuronal replacement as primary therapeutic mechanisms. Previous work with NSCs indicates that long‐term survival and integration with host tissue are not observed, and therapeutic effects may be linked to the paracrine activity. With this evidence, the focus switched to mesenchymal stem cells (MSCs), known for their paracrine and immunomodulatory potential (**Figure** **1**). They are readily obtainable from various sources such as bone marrow, adipose tissue, etc.^[^ ^1^ ^]^ MSC‐based treatments have demonstrated beneficial effects in different animal models of neurological diseases in experimental studies. At the same time, significant progress has been made in developing clinically accepted delivery and monitoring protocols. To date, 125 clinical trials applying MSCs to treat neurological diseases have been registered.

## 2 Experimental Attempts to Use Mesenchymal Stem Cells in the Treatment of Central Nervous System Diseases

Cell therapy using MSCs is currently one of the most dynamically developing branches of regenerative medicine. The simplicity of obtaining MSCs from various sources and their low immunogenicity and immunomodulatory abilities means that they can be transplanted in the auto‐ and allogeneic system. Also, the antiapoptotic, paracrine, and multidirectional ability of MSCs to differentiate has driven their current evaluation in translational research and clinical trials for the treatment of the most common diseases, including neurological disorders involving CNS structures such as stroke, Alzheimer's disease (AD), amyotrophic lateral sclerosis (ALS), Huntington's and Parkinson's diseases (HD, PD), multiple sclerosis (MS), and spinal cord injury, for which there are still no effective alternative treatments available (**Table** **1**).

**Table 1:** Summary of MSCs mechanisms of action and results obtained after their transplantation in CNS disorders

Preclinical studies	Stroke	Alzheimer's disease	Huntington's disease	Parkinson's disease	Amyotrophic lateral sclerosis	Multiple sclerosis	Spinal cord injury
Common MSC activity in CNS diseases							
Anti‐inflammatory activity	+	+	+		+	+	+
Disease symptoms amelioration					+	+	
Lesion reduction	+						+
Cellular death reduction/neuroprotection			+	+	+	+	+
Maintenance and remodeling of axons	+	+				+	+
Neuroprotective factors release	+	+			+		
Improvement of neurons’ functionality	+	+	+				
Functional improvement	+	+	+	+	+		+
Enhanced neurogenesis		+	+	+			
Pathognomonic protein deposits reduction		+	+				
Oligodendrogenesis stimulation						+	+
Remyelination						+	+
Blood vessels creation	+						+
Astrogliosis and microgliosis reduction	+	+			+	+	+
Prolonged lifespan			+		+		
Disease specific MSCs activity:							
Stroke	Protection of microvasculature against reperfusion injury BBB stabilization Brain edema reduction MSC‐derived mitochondrial transfer to endothelial cells						
Alzheimer's disease	Enhancement of pathological neurons autophagy						
Huntington's disease	Decreased brain atrophy						
Parkinson's disease	*α*‐Synuclein transmission inhibition						
Amyotrophic lateral sclerosis	Reduced motor neuron degeneration						
Multiple sclerosis	Stimulation of oligodendrocyte homing to lesion Postponed neurological dysfunction Autoantigen immunotolerance induction						
Spinal cord injury	Fibrosis reduction						

Clinical studies	Stroke	Alzheimer's disease	Huntington's disease	Parkinson's disease	Amyotrophic lateral sclerosis	Multiple sclerosis	Spinal cord injury
Safety	+	+		+	+	+	+
Reduction in mortality	+						
Improvement	+	−		+		+	+
Anti‐inflammatory activity	+				+		
Clinical stabilization				+		+	
Disease progression slowdown					+		

### 2.1 MSCs in Stroke

Stroke manifests in the form of sudden neurological deficits arising from the disruption of cerebral blood circulation. Due to the pathogenesis mechanism, there is an ischemic and hemorrhagic stroke. The occlusion of cerebral vessels characterizes the former, and their rupture causes the latter. Ischemic stroke is the most frequent brain disease. Pathologically, it consists of a necrotic core and surrounding penumbra. Depending on the stroke area, various symptoms may occur, such as loss of consciousness and coma, dementia, impairment in vision, sensation, speech, paresis of the lower and upper limbs, and facial nerve. The severity of disability in people after stroke may be mild, causing only a slight decrease in mobility, or may lead to severe impairment, where the patient requires continuous help. The only currently approved pharmacological method of treating ischemic stroke is the intravenous administration of tissue plasminogen activator to patients to dissolve the blood clot, causing the blood vessel's occlusion. However, the very narrow therapeutic window for carrying out this procedure, up to 4.5 h after the onset of stroke symptoms, limits the possibility of its use to a small percentage of patients (≈15%). In recent years, many papers showed the high efficiency of thrombectomy by the arterial route with a significant extension of the therapeutic window, including in some cases up to 24 hours (h).^[^ ^2^ ^]^ However, some patients still experience stroke despite the mechanical clot removal, although it is milder. In many centers, thrombectomy became a standard of stroke care, but it requires specialized equipment and highly trained specialists. Therefore, the availability of this treatment remains low in most countries around the world.^[^ ^2^ ^]^ Therefore, alternative or supplementary strategies for therapeutic results are warranted. One proposed method is stem cell‐based therapy. Among different types of stem cells, MSCs seem to offer the best prospects for stroke therapy. MSCs have been extensively investigated as a treatment in a variety of animal models of subacute, acute, or chronic stroke because of their neuroprotective and neurogenic potential and immunomodulatory function. In acute stroke, apart from neuronal death, the inflammatory reaction is upregulated, which destroys hypoxic tissue in the insult region and initiates cytokine cascades that enlarge the damaged area. The neuroprotective factor's transport and immunomodulatory ability of MSCs reduce the inflammation. In addition, delivery of MSCs in the chronic phase of stroke has been shown to activate regenerative mechanisms which can contribute to brain function restoration.

MSCs may be administered through intravenous (IV), and intra‐arterial (IA) routes, or intracerebral (IC) injection. After MSC infusion, a reduction of brain edema and lesion area was observed.^[^ ^3^, ^4^, ^5^, ^6^ ^]^ In different animal stroke models, MSC treatment demonstrated increased axonal density and remodeling around the ischemic lesions^[^ ^7^, ^8^, ^9^ ^]^ and was correlated with improved functional recovery.^[^ ^10^, ^11^, ^12^, ^13^, ^14^, ^15^ ^]^ The therapeutic effects of MSC transplantation could be attributed to the secretion of factors that promote axonal growth and neurogenesis.^[^ ^16^ ^]^ Recently, Nagahama et al. have shown that IV injection of MSCs enhanced cortical connections through the corpus callosum and enhanced the expression of synaptophysin in ipsilateral neurons in a rat model of cerebral infarction.^[^ ^14^ ^]^

Brain ischemia affects not only neurons but also other cell types, especially vascular cells. MSCs have been shown to promote angiogenesis and vasculogenesis by increasing blood vessel density and releasing different growth factors.^[^ ^17^, ^18^ ^]^ Moreover, engrafting MSCs protected injured cerebral microvasculature against ischemic–reperfusion injury. One of the key mechanisms of the beneficial effect may be mitochondrial transfer between exogenous MSCs and damaged endothelial cells.^[^ ^19^ ^]^ The therapeutic potential of MSCs in functional improvement after stroke may also depend on stabilizing the blood‐brain barrier (BBB). A decrease in BBB permeability in damaged neural tissue was observed following MSC infusion in different stroke models.^[^ ^14^, ^20^ ^]^ Recent studies revealed that transplanted MSCs interact with pericytes, astrocytes, and neurons, providing BBB integrity and maintenance.^[^ ^21^ ^]^

Stroke is accompanied by inflammatory and immune reactions, which have been activated at each stage of the disease. Therefore, neuroinflammation has been suggested as an attractive treatment target in stroke. Since MSCs demonstrate immunomodulatory properties, they are capable of dampening poststroke inflammatory processes. It was shown that MSC transplantation significantly reduced the local activation of astrocytes and microglia/macrophages and the influx of leukocytes, including T cytotoxic cells, to the brain insult.^[^ ^22^, ^23^, ^24^, ^25^ ^]^ Moreover, MSC infusion has been shown to play a part in the inflammatory cascade by diminishing the levels of proinflammatory cytokines: Interleukin (IL) ‐1*α*, IL‐1*β*, IL‐6, tumour necrosis factor (TNF)‐*α*, and chemokines while increasing the levels of anti‐inflammatory cytokines: IL‐4, IL‐10, interferon (INF)‐*β*.^[^ ^6^, ^24^, ^26^, ^27^, ^28^ ^]^

The positive results of MSC transplantation in preclinical stroke models provided a factual basis for clinical trials. Based on data obtained from the ClinicalTrials.gov website and the International Clinical Trials Research Platform site, MSCs were predominantly used in almost half of the clinical trials of cell therapy for stroke. According to the literature, MSCs were used in applications for the acute and chronic ischemic stroke phases. However, the detailed analysis revealed a trend to use MSCs in the acute phase of the disease since the primary mechanism of their action is to modulate the inflammatory response by immune regulation. Both autologous and allogeneic MSCs were studied, and the data were consistent with the safety of cell therapy for stroke patients.^[^ ^29^ ^]^ To date, 27 clinical trials applying MSCs to treat stroke have been registered.^[^ ^30^ ^]^ Initial human studies of MSCs after stroke focused on autologous cell therapies. The first clinical experiment was performed in 2005 in South Korea. Among 30 patients with cerebral infarct, five received IV infusion of autologous MSCs. Serial evaluation for one year showed no adverse cell‐related effects. Particularly noteworthy was the significant reduction in mortality within five years of stroke incidence compared to patients who did not receive MSC transplantation.^[^ ^31^ ^]^ The other Phase I studies also have demonstrated the safety and feasibility of autologous MSCs administered intravenously or intra‐arterially with modest improvements in recovery.^[^ ^32^, ^33^, ^34^, ^35^ ^]^ In Phase II, randomized multicenter trials, treatment with allogeneic MSCs infused IV was shown to be tolerated. Moreover, there were not observed immunological adverse events for allogeneic donor cells.^[^ ^36^, ^37^ ^]^ In clinical settings, the recipients of allogeneic MSCs demonstrated long‐lasting or transient neurological improvement. Additionally, allogeneic MSC infusion was associated with a short term decrease in circulating T cells and inflammatory cytokines.^[^ ^38^ ^]^ Only two randomized controlled Phase III trials with over 100 patients have been registered, but none has been completed. Allogeneic MSCs have become mainstream in use in the acute or subacute phase of stroke; however, recently, the Phase I/II study has been published of MSC infusion in patients with chronic stroke to evaluate allogeneic MSC therapy in this population. Intravenous transfusion of allogeneic bone marrow MSCs (BM‐MSCs) isolated from a single human donor into 36 patients was shown to be safe. There were seen improvements in functional status in some patients.^[^ ^39^ ^]^ Moreover, allogeneic, genetically modified BM‐MSCs (SB623) were implanted IC in 18 patients with stable chronic ischemic stroke. The implantation of SB623 to the sites surrounding the subcortical stroke region was safe and accompanied by improvements in neurological recovery in 12 patients in a 2‐year Phase I/II study.^[^ ^40^ ^]^

Based on the experimental studies, MSCs derived from blood, bone marrow, or abdominal fat tissue, transplanted IV, IA, or IC in different stroke models ameliorate neurological deficits in graft recipients. Infusion of MSCs facilitates functional recovery via promoting neurogenesis and neural differentiation, stimulating angiogenesis and vasculogenesis, displaying immunomodulation of immune reaction accompanying a stroke. All clinical studies reported no detrimental effects due to MSC therapy. Some patients in cell treatment groups showed neurological recovery comparing with controls.

### 2.2 MSCs in Traumatic Brain Injury

Traumatic brain injury (TBI) is the most severe disease with high incidents among young individuals. It usually occurs by the head's external mechanical forces, leading to impaired neurological functions and even death. The damage caused by TBI can be divided into two phases. The early stage is the initial insult's immediate effect, leading to BBB disruption, brain swelling, and cranial hemorrhage.^[^ ^41^ ^]^ Oxidative stress and excitotoxicity in the acute stage of the disease result in rapid cell death within a localized or diffused brain area.^[^ ^42^ ^]^ The next phase is the second injury activated by prime injury 1–3 days after the initial traumatic episode, which extends for weeks or months. Progressive secondary damage is related to the release of excitatory amino acids, ionic imbalance, calcium overload, mitochondrial dysfunction, and it causes ongoing neurodegeneration.^[^ ^43^ ^]^ The severity of TBI's secondary mechanisms involves cell death, axonal damage, and diminished neurogenesis.^[^ ^44^ ^]^ Moreover, immunological and inflammatory responses accompanying brain injury extend neuronal damage. Post‐traumatic neuroinflammation is characterized by secretion of pro‐inflammatory cytokines, immune cell recruitment, and microglial activation.^[^ ^45^ ^]^

To date, no single treatment approach is effective for reducing TBI mortality or improving patients' functional recovery. A variety of pharmacological drugs did not ameliorate the outcome of the disease. Monotarget therapy for TBI was not effective due to the wide variety of factors occurring during the disease onset. Therefore a multitarget therapeutical strategy is needed. One promising option is cell transplantation. Many preclinical studies indicate that MSC application in different experimental models of TBI can cope with multiple disease pathology aspects.

Direct infusion of MSCs into injured brain or IV or IA delivery attenuated TBI‐induced motor and cognitive deficits in animals.^[^ ^46^, ^47^, ^48^ ^]^ The experimental data showed that the treatment with MSCs stimulated the injured brain to induce trophic factors contributing to promoting neurogenesis, neuroprotection, and neural repair in TBI rats and mice.^[^ ^49^, ^50^, ^51^ ^]^

MSC therapies have more advantages in modulating inflammation. Lin and co‐workers demonstrated that MSC transplantation downregulated proinflammatory genes and upregulated anti‐inflammatory genes in TBI rats' brains.^[^ ^48^ ^]^ Immunomodulatory properties of MSCs manifest in reprogramming microglia from proinflammatory (M1) to anti‐inflammatory (M2) phenotype of TBI recipients.^[^ ^52^, ^53^, ^54^ ^]^

It was shown that the genetic modification of MSCs improves their therapeutic effect after transplantation in TBI animal models. Genetically engineered MSCs that overexpress fibroblast growth factor 21 (FGF‐21), injected IC into TBI mouse brain enhanced cell homing to the injury site and increased hippocampal neurogenesis.^[^ ^55^, ^56^ ^]^ Similarly, Shi and co‐workers indicated that MSCs transferred with CXC chemokine receptor 4 (CXC‐R4) improved the migratory ability of MSC‐CXC‐R4 in TBI rats.^[^ ^57^ ^]^ The promising approach of using genetically modified MSCs for TBI treatment is to overexpress anti‐inflammatory factors. Recent studies showed that genetically altered MSCs to overexpress IL‐4 or IL‐10 infused in experimental models of TBI protected neural cells from inflammation effects. They also promoted microglia to express M2 phenotypic markers and reduce the production of TNF‐*α* in the injured brain.^[^ ^58^, ^59^ ^]^ It is known that hypoxia complicates TBI contributing to secondary brain injury. Genetically modified MSCs to overexpress hypoxia‐inducible factor 1*α* (HIF‐1 *α*) revealed more remarkable improvement in TBI mice's neurological recovery compared with native MSC transplantation.^[^ ^60^ ^]^

It is well documented that the therapeutic potential of MSCs is related to the secretion of bioactive factors. Recent findings indicate that exosomes released from MSCs exhibit an effect similar to their counterparts. Exosomes could be administrated IV, IA, intracerebroventricularly (ICV), intrathecally (IT) or IN. Cell‐free exosomes derived from human MSCs have been studied in vivo in experimental animals subjected to TBI. Intravenous injection of MSC exosomes to TBI rats significantly ameliorates motor deficits and improved spatial memory by promoting endogenous neurogenesis and angiogenesis.^[^ ^61^, ^62^ ^]^ Recently, the secretome of MSCs infused IV was shown to alleviate neuroinflammation, limiting the secretion of proinflammatory cytokines and modulating microglia polarization, and ameliorating neural cell loss.^[^ ^63^, ^64^ ^]^ In another study, IC, transplantation of MSC‐derived exosomes in TBI rats prevented microglia proinflammatory activation, thereby alleviating neural injury and facilitating functional recovery after brain insult.^[^ ^65^ ^]^ Exosomes isolated from MSCs have also been reported to diminish neurological injury in TBI's large animal model. Williams and co‐workers demonstrated that exosomes derived from human BM‐MSCs infused IV in swine subjected to severe TBI improved BBB integrity, decreased brain swelling, and lesion site.^[^ ^66^, ^67^ ^]^

The results from experimental studies provide a promising approach for the clinical application of MSCs in TBI patients. To date, a relatively small number of clinical trials with MSC therapy for TBI exist. Autologous BM‐MSCs transplanted into the injured brain during cranial operation in TBI disorders have shown no adverse effects.^[^ ^68^ ^]^ Similarly, administration of autologous BM‐MSCs via lumbar puncture of 97 patients in the subacute stage of TBI was proved to be safe and efficient. Approximately 40% of patients showed improved neurological function following MSC transplantation.^[^ ^69^ ^]^ In Phase, I trials, autologous BM‐MSC delivery was reported to decrease neural cell loss, neuroinflammation depletion, and improved clinical outcomes after TBI in adults and children.^[^ ^70^, ^71^ ^]^

In summary, the results of experimental and clinical studies demonstrate that transplantation of MSCs in TBI recipients enhances neural tissue repair by stimulation of neurogenesis, angiogenesis, maturation of newborn neurons and their neuroprotection, and modulation of the inflammatory processes in the injured brain. It improves cognitive and motor functional recovery and reduces brain tissue damage in TBI disorders.

### 2.3 MSCs in Alzheimer's Disease

AD is a chronic neurodegenerative disease that manifests itself as progressive dementia resulting in memory loss and cognitive impairments. The histopathological picture of the brain in AD disorders shows the accumulation of amyloid *β* (A*β*) plaques and intracellular formation of neurofibrillary tangles that lead to loss of cholinergic neurons. It is also established that neuroinflammation plays a significant role in AD.^[^ ^72^ ^]^ Chronic accumulation of A*β* activates microglia, which accelerates neuronal loss and cognitive decline.^[^ ^73^ ^]^ Among all treatment approaches, MSC‐based therapy is ready to apply modality for AD. Experimental studies of animal models of AD, IV, IC, and intraventricular (INVE) infusions of MSCs have been performed.

Preclinical studies indicate that MSC infusion improves cognitive impairments in AD recipients. The rescue of memory deficits has been reported by reducing A*β* deposition, provoking its clearance in AD‐treated animal models.^[^ ^74^, ^75^ ^]^ Human MSCs have been shown to decrease the levels of A*β* by enhancing autophagy of pathological neurons in an AD mice model.^[^ ^76^ ^]^ Others have reported enhanced neurogenesis in the hippocampus of A*β*‐treated mice or rats transplanted with MSCs by enhancing cell proliferation and Nestin expression followed by the presence of sex determining region Y‐box 2 (SOX2), and NeuroD indicated neuronal differentiation and diminishment of long term survival of newly generated neurons.^[^ ^77^, ^78^, ^79^, ^80^ ^]^ It was shown to be accompanied by upregulation of the levels of neurotrophic factors, i.e., brain‐derived neurotrophic factor (BDNF), nerve growth factor (NGF), and vascular endothelial growth factor (VEGF) in the brains of AD disorders receiving MSC graft that could protect neurons and neuronal integrity.^[^ ^81^ ^]^ Infusion of MSCs in the brain in an AD rat model increased the level of acetylcholine and the expression of choline acetyltransferase and acetylcholinesterase, which are the proof of functional improvement of hippocampal neurons.^[^ ^82^, ^83^, ^84^ ^]^ Cognitive impairment in AD also is related to synaptic loss. In recent studies, Zappa Villar et al. observed that MSC treatment restored the levels of Synaptotagmin‐1 (SYT1), Synaptophysin (SYP), and glutamic acid decarboxylase 65 synaptic markers in the hippocampus of the sporadic AD rat model, suggesting a protective role of MSCs against synaptic protein loss.^[^ ^85^ ^]^ Moreover, since it was shown that microglial processes have connections with neuronal synapses, prolonged activation of microglia related to AD induce synaptic toxicity, and accelerates neuronal loss.^[^ ^86^ ^]^

It has been revealed that MSC infusion can change the inflammatory effect in AD animal models. Significant reduction in microglial activation in mouse mice cortexes and decreased expression of proinflammatory factors, i.e., TNF‐*α*, IL‐6, Macrophage chemotactic protein (MCP)‐1, was observed in MSC recipients.^[^ ^87^, ^88^ ^]^ Transplantation of MSCs has been shown to switch activated microglia from M1 phenotype producing proinflammatory cytokines to M2 phenotype, which have an anti‐inflammatory effect on AD and improve neuron survival.^[^ ^89^, ^90^ ^]^

Numerous clinical trials in patients with AD have been registered based on the experimental results using MSCs in A*β*‐treated animals.^[^ ^91^ ^]^ In the first Phase I clinical trial in Seoul, South Korea, in 2011, nine patients with mild/moderate AD‐induced dementia underwent stereotactic brain infusion of allogeneic MSCs (NCT01547689). The administration of MSCs was shown to be a feasible and well‐tolerated method. Subsequent Phase I/II clinical trials with allogeneic MSCs transplanted into the hippocampus of AD patients in South Korea were also reported to be safe. No patients showed serious complications during 18–24 months of follow‐up (NCT01696591 and NCT02054208). In 2015, the food and drug administration (FDA) accepted the first Phase II trial of MSCs for AD treatment in the United States (U.S.). The multicenter, placebo‐controlled study enrolling 40 patients with Alzheimer's dementia was carried out in California, and the assessment of activities using the national institutes of health (NIH) cognitive scale has been applied (NCT02833792). Now similar trials are running in other centers in Europe and Asia; however, no significant improvement in the clinical status of AD patients treated with MSCs was observed.

Numerous studies have shown that AD patients' treatment using different pharmaceuticals only relieves the symptoms without curing the disease. Recently, stem cell therapy, predominantly MSC transplantation, provides new potential in the treatment of AD. Based on preclinical research, MSCs have been shown to decrease A*β* deposits and abnormal protein degradation, upregulate acetylcholine levels and increase neuronal survival, thereby improving spatial learning memory of AD animal models. Moreover, MSC transplantation enhances hippocampal neurogenesis and stabilizes synapses in rodents subjected to AD. Importantly, MSC infusion regulates neuroinflammation by adjusting microglia and astrocytes' activation in AD disorders' brains. The improvement of AD symptoms observed in experimental studies maintains excellent prospects for MSC therapy in AD patients. Single trials with stereotactic brain infusion of MSCs in AD patients demonstrated to be feasible and safe without serious complications.

### 2.4 MSCs in Huntington's Disease

Huntington's disease occurs due to a mutation in a gene encoding a protein called huntingtin, accumulating in excessive amounts in cells and has a cytotoxic effect. During HD, brain neurons die off, particularly neurons located within the striatal structures such as the caudate nucleus and crust, secreting *γ*‐aminobutyric acid. The disease's symptoms usually appear around 30–40 years of age and are associated with impaired motor function, behavior, and cognitive ability. At present, there is no effective treatment for slowing HD progression. The therapeutic effect of MSCs was reported in animal models of HD.^[^ ^92^, ^93^, ^94^ ^]^ For most MSC transplant studies conducted to date in HD, exogenous cells were administered intracerebrally. HD mice treated with BM‐MSC showed decreased motor deficits and an improvement in spatial memory.^[^ ^95^, ^96^ ^]^ Transplanted BM‐MSCs stimulated endogenous neural stem cell proliferation, probably by inducing trophic support with increased BDNF levels in the striatum of HD mice.^[^ ^97^, ^98^, ^99^ ^]^ The HD mice treated with genetically engineered MSCs overexpressing BDNF or NGF factors revealed the reduction of apoptotic cells in the striatal region and decreased brain atrophy.^[^ ^100^ ^]^ Furthermore, in experimental models of HD receiving MSC transplantation, the number of misfolded forms of huntingtin protein (m HTT) aggregates was diminished, and HD disorders' lifespan was prolonged compared with control mice.^[^ ^101^ ^]^ Recent studies suggest that m HTT aggregates could be transferred from the host neurons to the donor cells, inducing spreading neurodegeneration in the brain.^[^ ^102^ ^]^

In addition to the intracranial injection of MSCs, intranasal cell administration was performed in an HD mouse model. Treated mice had a regular sleep cycle and increased survival time compared to untreated animals, who showed disrupted circadian rhythms and shorter life spans.^[^ ^103^ ^]^ The authors have shown that autologous BM‐derived MSCs infused intranasally resulted in increased striatal expressions of tyrosine hydroxylase (TH) and a phosphoprotein related to the dopamine D1 receptor (DARPP‐32) proteins involved in the dopamine signaling cascade. MSC treatment also revealed immunomodulatory effects by microglial morphology alteration into M2 anti‐inflammatory subtype and suppressing proinflammatory gene expression of TNF‐*α*, IL‐6, and MCP‐1, which is usually upregulated in the brain of HD mice.

Clinical treatment for HD has been incredibly challenging due to the complex symptomatology of the disease. It has been demonstrated that HD patients have low levels of BDNF responsible for cortical neurons' survival and function.^[^ ^104^ ^]^ Restoration of BDNF level in transgenic HD rodent models improves neuronal survival and ameliorates HD symptoms. Therefore BDNF could be considered for the treatment of neuronal dysfunction observed in HD patients. However, experimental studies were shown that direct injection of BDNF has been ineffective in HD disorders because of the short half‐time of protein. Therefore the proposed clinical trial in HD patients was appointed to transplant genetically engineering MSCs overexpressing BDNF (NCT01937923).^[^ ^105^ ^]^

Based on experimental studies, MSC treatment revealed improved motor and cognitive deficits in HD's mouse and rat models. Human MSCs transplanted in HD disorders have decreased atrophy observed in the striatum and HTT aggregates as well as stimulated endogenous neurogenesis and extended life span. The positive results obtained from preclinical studies suggested that MSC therapy may be attractive for HD patients.

### 2.5 MSCs in Parkinson's Disease

The progressive degeneration of dopamine‐producing neurons found in the brain substantia nigra leads to the development of PD. The disease's main symptoms include resting tremor, muscle stiffness, bradykinesia, problems with posture, and limited ability to control precise movements. Also, there is impairment of cognitive functions, sleep, and smell disorders. At present, the causative agents of the disease are unknown. In most cases, it develops spontaneously; however, about 5% of patients have mutations in the gene encoding *α*‐synuclein, which is associated with the formation of protein aggregates in the bodies of neurons called Lewy bodies, which have a cytotoxic effect.^[^ ^106^ ^]^ In animals in the PD model, administration of MSCs was mainly intra‐cerebrally, but intravenous, intra‐arterial, and intranasal administration was also attempted.

In research studies, transplantation of MSCs has been shown to improve impaired motor functions caused by PD. Systemic infusion of human MSCs in rat PD disorders reduced uncoordinated limb movement measured in behavioral tests.^[^ ^107^, ^108^ ^]^ It was associated with elevated dopamine levels in the striatum of MSC recipients and an increased number of tyrosine hydroxylase (TH)‐positive dopaminergic neurons, suggesting a regenerative effect of MSC. Similarly, direct striatal injection of MSCs in rodent models of PD improved locomotor activity, enhanced neurogenesis, and induced neuroblast migration.^[^ ^109^, ^110^, ^111^, ^112^ ^]^ MSC treatment has been reported to inhibit transmission of *α*‐synuclein in a Parkinsonian model.^[^ ^113^ ^]^ Moreover, the antiapoptotic factor B‐cell lymphoma 2 (Bcl2) was observed to be upregulated. By contrast, the expression of proapoptotic factor Bcl‐2‐associated X‐protein (Bax) after MSC transplantation was decreased in PD mice, suggesting a cell‐protective effect in neurodegeneration.^[^ ^114^ ^]^

Previous studies revealed that inflammation is associated with PD, including astrogliosis and microgliosis.^[^ ^115^, ^116^ ^]^ In PD, a strong correlation between the disease and Glial fibrillary acidic protein (GFAP) values in peripheral blood has been found.^[^ ^117^, ^118^ ^]^ MSC infusion decreased gene expression of GFAP in venous blood of the rat model of PD and reduced microglial activation.^[^ ^108^ ^]^ Adipose‐derived MSCs transplanted in a rat model of PD upregulated peripheral anti‐inflammatory cytokines.^[^ ^111^ ^]^

It was shown that the activation of MSCs before transplantation revealed a more pronounced effect in PD disorders by preconditioning of MSCs by curcumin protected neurons from apoptosis in PD models in vitro and in vivo.^[^ ^114^, ^119^ ^]^ Similarly, genetically engineered MSCs with different neurotrophic factors, i.e., BDNF, Cerebral dopamine neurotrophic factor (CDNF), hepatocyte growth factor (HGF), Neurotrophin‐3, demonstrated increased dopaminergic neurons and enhanced motor recovery after their injection in experimental PD.^[^ ^115^, ^120^ ^]^

There are not much data available on MSC transplantation in clinical trials of PD. In the procedure related to cell therapy in PD patients, stereotactic injection of MSCs into the brain or systemic and intranasal infusion has been performed. Autologous BM‐MSCs injected into the subventricular zone of seven patients with PD appeared to be safe and well‐tolerated, with long‐lasting motor function improvement observed in some patients.^[^ ^121^ ^]^ In another clinical study, five patients affected by progressive supranuclear palsy, a rare, severe form of Parkinsonism, received BM‐MSCs by infusion into the cerebral arteries. In these patients, in whom deterioration of motor function is invariably rapid, clinical stabilization for at least six months was observed.^[^ ^122^ ^]^ Allogeneic BM‐MSCs or adipose tissue MSCs transplanted in idiopathic PD patients induced favorable changes in disability as measured by the unified PD rating scale. They manifested an improvement in facial expression and gait. Moreover, functional connectivity between the substantia nigra and striatum was visualized by MRI.^[^ ^123^ ^]^

In the context of the referred studies, MSC application for PD treatment significantly improves motor behavior in animal models of the disease. It was shown that MSCs survive in the transplanted area for a couple of weeks after IC infusion in rodent models of PD. Exogenous MSCs secrete a broad spectrum of factors that reveal immunomodulatory properties, inhibit apoptosis, promote neuronal survival, and differentiate PD mice and rats. After MSC transplantation to the brain of PD animals, TH level and the number of DA neurons were observed in the damaged area. The preliminary data from clinical trials revealed that MSC application in PD patients is well tolerated and improves motor functions being a promising method for PD treatment.

### 2.6 MSCs in Amyotrophic Lateral Sclerosis

ALS is a disease of undefined etiology that usually occurs sporadically with a rapidly fatal course three to five years after symptom onset. During ALS, progressive degeneration of the upper and lower motor neurons in the brain and spinal cord results in muscle weakness and respiratory failure.

In experimental models of ALS, the administration of MSCs was shown to alleviate disease symptoms. Neuroprotection, stimulation of nerve tissue regeneration, and increased lifespan have been observed in ALS disorders after MSC transplantation.^[^ ^124^, ^125^, ^126^ ^]^ Transplanted MSCs may act as bystander cells secreting neurotrophic factors distributed via cerebrospinal fluid (CSF) from the motor cortex to the spinal cord. The positive effects of MSC administration in ALS are most likely related to the neuroprotective effects of factors secreted by transplanted MSCs such as NGF, BDNF, Insulin‐like growth factor (IGF)‐1, and VEGF.^[^ ^127^, ^128^, ^129^ ^]^ Nakanishi et al. proved that the infusion of modified MSCs simultaneously expressing GDNF, IGF‐1, and HGF significantly improved the neurotrophic effect of donor cells and delayed onset of symptoms in a mouse model of ALS.^[^ ^130^ ^]^

Intrathecal infusion of MSCs counteracted the development of neurodegenerative changes related to ALS. The results of different studies indicated the improved motor function of MSC recipients in ALS models and reduced motor neuron degeneration by protecting the structure of altered perineuronal nests.^[^ ^124^, ^131^, ^132^ ^]^ Recently, Kook et al. observed positive effects of MSCs transplanted intramuscularly into a mouse model of ALS. Repeated injections of human umbilical cord blood MSCs into gastrocnemius muscles of superoxide dismutase 1 (SOD1) G93A mice ameliorated muscle atrophy and the rate of neuromuscular degeneration in skeletal muscles leading to the increased motor function of cell graft recipients and prolonged lifespan.^[^ ^133^ ^]^

Experimental studies showed that MSCs transplanted in ALS also possess an anti‐inflammatory function by reducing astrogliosis and microgliosis and decrease peripheral levels of proinflammatory cytokines such as TNF‐*α*, IL‐1, IL‐6 in CSF.^[^ ^134^, ^135^, ^136^, ^137^ ^]^

Clinical studies conducted in ALS patients treated with MSCs showed that, as in experimental animals, patients had a slowdown in disease progression. Phase I/II clinical trials revealed that intrathecal administration of bone marrow or adipose tissue‐derived MSCs were safe and well‐tolerated.^[^ ^79^, ^138^, ^139^, ^140^ ^]^ In some studies, MSC treated patients displayed a slight decline in ALS disease progression.^[^ ^141^, ^142^ ^]^ Postmortem evaluation of ALS patients treated with MSCs showed that a more significant number of motor neurons were preserved at the height of the spinal cord area where the cells were administered, compared to other spinal sites.^[^ ^140^, ^143^, ^144^ ^]^ Observed clinical improvement in the post‐transplantation ALS recipients might also be related to MSCs' immunomodulatory effect. Recently, Phase II randomized controlled clinical trial was conducted in 48 ALS patients treated with autologous genetically modified MSCs overexpressed neurotrophic factor (NTF) delivered via intramuscular and IT injection. The positive response to MSC/NTF injection was seen in biomarker evaluation in neurospinal fluid collected from ALS treated patients. The decrease of proinflammatory markers, i.e., stromal cell‐derived factor 1 (SDF‐1) and MCP‐1, and the increase of neurotrophic biomarkers, i.e., leukemia inhibitory factor (LIF), HGF, VEGF, micro RNA (miR)‐132p, and miR‐146 were observed in the CSF of ALS patients subjected to MSC/NTF therapy.^[^ ^145^ ^]^ The enrollment of 261 ALS patients in randomized, double‐blind, placebo‐controlled Phase III trials was completed. The study aims to estimate the safety and effectiveness of three MSC/NTF infusions administered IT to ALS patients every two months. The neurological patient score and biomarkers assessment will be evaluated in blood and CSF of ALS recipients subjected to MSC/NTF transplantation (NCT03280056). The latest case‐control study involved 67 ALS patients treated with MSCs derived from Wharton's jelly (WJ‐MSCs). All patients received three IT infusions of Wharton‐s jelly (WJ)‐MSCs. In the whole study population, a decrease in the disease's progression was observed in 31% of patients. The early response to WJ‐MSC treatment predicted the outcome in ALS patients and extended the survival. The results of this medical experiment are encouraging; the authors stated that in the future time it is worthwhile to investigate the additive effect of pharmaceuticals to MSC therapy in ALS patients.^[^ ^146^ ^]^

The current pharmaceutics approved by FDA for ALS treatment demonstrated only slightly reduced functional impairment and a modest survival benefit in disease disorders. Over the last several years, MSC therapy was proposed for ALS treatment based on preclinical studies' positive results. Transplanted MSCs act as bystander cells, scavenging toxic substances and secreting trophic factors, which contribute to the survival and neuroprotection of neural cells observed in different motor neuron models. Infusion of MSCs into ALS rodents ameliorates motor symptoms and improves the life‐span of animals. Intrathecal administration of MSCs in experimental SOD1 mutant mice induced proliferation of endogenous neural progenitor cells and modulated local inflammation. Clinical studies indicate that autologous or perinatal MSCs injected in ALS patients reduced disease progression in some individuals.

### 2.7 MSCs in Multiple Sclerosis

MS is an inflammatory and demyelinating autoimmune disease of the CNS. The disease's pathophysiology is associated with the formation of autoreactive lymphocytes and antigen‐presenting cells in the body. These cells' inflammatory cytokines promote the recruitment of elements of the immune system such as macrophages, mast cells, neutrophils, and lymphocytes to the CNS structures associated with astrogliosis and microgliosis. Neuroinflammation related neurodegeneration in the chronic phase of MS destroys myelin sheaths and axon neurons, leading to multifocal degenerative changes visible in the form of MS—areas of demyelination and massive loss of neurons. The clinical picture of a patient with MS is highly dependent on the location of the lesion. However, there is usually paresis, visual impairment, sensation, speech, balance, coordination, memory problems, and cognitive decline.

In the animal model of the disease—experimental allergic encephalomyelitis (EAE), the systemic administration of MSCs induces tolerance formation in the transplant recipient for its antigens. In the spinal cord area, increased oligodendrogenesis and stronger remyelination than control animals were observed in MSC transplanted animals, reducing the invasion of autoaggressive leukocytes in the brain. Infusion of MSCs into EAE mice decreased the degree of local inflammation in the CNS.^[^ ^147^, ^148^, ^149^, ^150^ ^]^ The anti‐inflammatory effect of MSCs is beneficial for neuroprotection, prevents axon loss, and reduces neuronal necrosis and apoptosis in the brain cortex and spinal cord in EAE.^[^ ^151^, ^152^ ^]^ Transplanted MSCs inhibited demyelination and stimulated oligodendrogenesis with newly formed myelin sheaths surrounding axons visible in the corpus callosum and spinal cord of acute EAE disorders.^[^ ^153^, ^154^ ^]^ This results in a much milder course of the disease with a lower incidence of relapse, a reduction in the number of immune cells infiltrate, and a decrease in demyelination and axonal loss.^[^ ^155^, ^156^ ^]^ Recently, MSC therapy was performed in a primate MS model. Intrathecal infusion of MSCs postponed neurological dysfunction and neuronal demyelination in EAE monkeys.^[^ ^157^ ^]^

In a cuprizone (CPZ) induced mouse model of chronic MS, intracerebral MSC transplantation diminished neuroinflammation by reducing activation of astrocytes and microglia as well as shifting proinflammatory subtypes of microglia (M1) into anti‐inflammatory microglia (M2) in the host brain.^[^ ^158^ ^]^ Additionally, MSC treatment‐induced improvement in remyelination and axonal recovery as visualized in immunohistochemical and transmission electron microscopy studies. This observation is in line with the previous studies where MSC infusion in a chronic demyelination mouse model of MS revealed oligodendrocyte progenitor cell migration, homing in the injured area, and enhanced myelinated fibers detected in the corpus callosum.^[^ ^159^, ^160^ ^]^ Interestingly, preconditioned MSCs with SDF‐1 responsible for cell chemotaxis to the site of injury, delivered intranasally into CPZ‐induced mice reduced protein expression of astrocyte GFAP and microglia Iba‐1markers and increased oligodendrocyte factor Oligodendrocyte transcription factor (Olig)‐2 in the brain.^[^ ^161^ ^]^ Infusion of SDF‐1 preconditioned MSCs improved spatial learning and memory deficit observed in a non‐treated CPZ‐induced chronically demyelinated mice model of MS.

Several clinical studies using MSC transplantation in MS have been performed. Most clinical trials involved a limited number of subjects with autologous BM‐MSCs infused intrathecally or intravenously. The first pilot study was performed in Iran in 2007.^[^ ^162^ ^]^ Based on the literature analysis, 23 clinical trials in MS patients with autologous or allogeneic MSCs derived from BM, adipose tissue (AD), and umbilical cord (UC) MSCs have been registered. The numerous reports concern early‐stage (Phase I/II) clinical trials.^[^ ^163^, ^164^, ^165^ ^]^ The procedure of IT or IV MSC transplantation proved feasible, safe, and tolerable. Some patients showed signs of clinical stabilization or an improvement measured by an expanded disability status scale.^[^ ^166^, ^167^ ^]^ Immunomodulatory effect of MSCs was confirmed by an increase of the levels of anti‐inflammatory cytokines, i.e., IL‐4, IL‐10, and trophic factors, i.e., interferon *γ* (IFN‐*γ*) and HGF in peripheral blood of MS patients after MSC infusion.^[^ ^130^ ^]^ Recently, the study protocol has been established for randomized, double‐blind, cross‐over Phase I/II clinical trials with autologous BM‐MSCs for the therapy of MS.^[^ ^168^ ^]^ The studies were performed in several national trials. The results of the study have not been published yet. Similarly, a Phase I clinical study was conducted on 7 MS patients in Sweden. The IV injection of autologous BM‐MSCs transplanted during clinical remission stabilized disability status in 86% of MS patients. The increased proportions of regulatory T lymphocytes in peripheral blood detected in one week after infusion point to the immunotolerogenic effect of MSCs in MS patients.^[^ ^169^ ^]^ The analyses of main outcomes in MSC treated MS patients have been proposed to explore microstructural tissue integrity using MRI.^[^ ^170^, ^171^ ^]^

Therapies of MS disorders mainly focus on diminishing inflammation; therefore, cell‐based therapy using MSCs that manifest immunomodulatory properties are promising in MS treatment. Based on preclinical studies, transplantation of MSCs modulates immune response accompanying MS disease. By secretion of different neurotrophic factors and anti‐inflammatory cytokines, transplanted MSCs maintain a favorable microenvironment to reduce microglial activation and promote neuroprotection. It was demonstrated that MSC infusion in MS animal models induces oligodendrogenesis and stimulates remyelination and nerve conduction velocity. Autologous MSCs infused into progressive MS patients resulted in a mild improvement of neurological disability in some individuals. The immunomodulatory effect of MSCs was confirmed by the suppression of dendritic cells and T1 lymphocytes and induction of switch M1 phenotype to M2 microglia and an increase in anti‐inflammatory levels cytokines in MS patients after MSC infusion.

### 2.8 MSCs in Spinal Cord Injury

Mechanical, accidental spinal cord injury (SCI) leads to disruption of the neural motor and sensory tract, resulting in permanent disability, significantly impacting patients' quality of life. The primary pathological changes arising after spinal injury include disruption of axons and blood vessels. Still, secondary changes include disturbance of local ionic concentrations, loss of blood pressure regulation, reduced blood flow through the spinal cord, disruption of the BBB, cell activation immune response, cell apoptosis, and excitotoxicity lead to patients' deterioration and hurt the regeneration process. Inhibition of these processes is a potential target for stem cell transplant therapy. Increased angiogenesis, oligodendrocyte proliferation, axonal regeneration and re‐myelination, reduction of fibrosis, and postinjury lesion were observed after MSC transplantation. The beneficial effect of MSC transplantation in SCI has been shown in different experimental studies after intrathecal, intracerebral, or intravenous cell infusion. However, it was observed that the therapeutic competence of MSCs increase more efficiently if they are infused into the site of injury. Transplanted MSCs exerted a neuroprotective function against cell death and increased the proportion of intact tissue in SCI of rats.^[^ ^172^, ^173^, ^174^ ^]^ Intrathecally grafted MSCs resulted in a higher amount of white matter and improved axonal sprouting in the lesion area of the spinal cord with a high expression of genes related to axonal growth factors.^[^ ^132^ ^]^ This positive impact of MSCs resulted in the restoration of motor and sensory functions in SCI models.^[^ ^175^, ^176^, ^177^, ^178^, ^179^ ^]^ Furthermore, it was demonstrated that MSCs seeded in different scaffolds transplanted into injured spinal cord facilitate post‐traumatic regeneration of nervous tissue to a greater extent than MSCs infused as a cell suspension.^[^ ^180^, ^181^, ^182^ ^]^

In addition to the neuronal restoration in SCI models, MSC transplantation reveals an immunomodulatory effect and lessening the disease's pro‐inflammatory reaction. It was shown that administration of MSCs reduces microglia and astroglia activation at the site of spinal cord injury.^[^ ^183^, ^184^, ^185^, ^186^ ^]^ In MSC‐treated SCI rats, the level of proinflammatory cytokines IL‐1*β*, IL‐6, TNF‐*α* was decreased, but anti‐inflammatory cytokines IL‐10 and IL‐12 were increased.^[^ ^187^, ^188^ ^]^

The first of the pilot attempts to use MSCs in the treatment of postaccident, incomplete, and complete spinal cord rupture in humans yielded positive results. The degree of response of individual patients to the administration of MSCs varied; however, the majority observed an improvement in the clinical picture. Of the 26 clinical studies registered for SCI patients, MSCs isolated from bone marrow, adipose tissue, or umbilical cord were used. MSCs have been transplanted intrathecally and intravenously. The results of Phase I/II clinical trials demonstrated that MSC infusion in SCI recipients was safe and well‐tolerated. MSC transplantation positively affected motor function, including improving upper limb motility and sensation within the damaged area in people with tetraplegia confirmed by neurophysiological studies. Relative to rehabilitation therapy, a reduction in neuropathic pain and sensory and bladder function improvements were reported. In many categories, the patients' condition was reclassified from severe to moderately severe and from moderately severe to mild, respectively. Despite the clear functional improvement in patients assessed according to the American Spinal Injury Association Scale and International Association of Neurorestoratology‐Spinal Cord Injury Functional Rating Scale, neuroimaging studies did not show changes in morphological images or signs of regeneration of damaged spinal cord.^[^ ^189^, ^190^ ^]^ Recently, a multidisciplinary Phase I clinical trial with intrathecal administration of adipose tissue‐derived MSCs in SCI patients is ongoing at the Mayo Clinic. The first report from this trial has shown clinical signs of efficacy in one patient observed at 3, 6, 12, and 18 months after MSC transplantation, suggesting improved rather than stabilized status of the host.^[^ ^191^ ^]^

Based on preclinical studies, human MSCs transplanted in animal models of SCI ameliorate the deleterious proinflammatory reaction. It contributed to the reduction of microglia and astrocyte activation at the site of injury. Injection of MSCs into the injured spinal cord exerts neuroprotective and neurotrophic effects promoting neuronal regrowth and restore motor and sensory tasks in SCI rodents. In the clinical trials, MSC transplantations were performed during acute, subacute, or chronic SCI patients. Intrathecal administration of autologous or allogeneic MSCs turned out to be well‐tolerated, and clinical improvement manifested in sensory and motor recovery have been observed in some SCI patients.

To resume, transplantation of native or genetically engineered MSCs isolated from different sources or secretory cell products can manage multiple neural disease pathology aspects. The data obtained from experimental and clinical studies identify several molecular mechanisms through which MSCs perform their therapeutic activity. Preclinical studies revealed that MSCs or their derivate transplanted in animal models of CNS diseases display immunomodulatory and anti‐inflammatory functions. Notably, neuroprotection and nerve regeneration has been demonstrated in various studies after MSC infusion in rats and mice exhibiting the neural deficits. Transplanted MSCs were shown to decrease neural damage and improve functional loss, enhancing animals' behavioral activities. Clinical translation MSC therapy in neurological disorders progresses relatively slowly despite encouraging results demonstrated in animal models. However, the administration of MSCs in patients with neural disabilities showed satisfying efficacy in specific clinical trials (**Table** **2**). Nevertheless, further studies to improve the effectiveness of MSC transplantation to treat disabilities of CNS are necessary.

**Table 2:** Qualitative stratification of transplanted MSCs effect size in neurological disorders ranging as: 1) no improvement, 2) unknown, 3) symptoms alleviation, 4) significant improvement, and 5) full recovery

MSCs’ effect size stratification	Animal models	Clinical Trials	No. of clinical trials
Stroke	Significant improvement	Significant improvement	26
Traumatic brain injury	Significant improvement	Significant improvement	3
Alzheimer's disease	Significant improvement	No improvement	14
Huntington's disease	Symptoms alleviation	Unknown	3
Parkinson's disease	Significant improvement	Significant improvement	8
Amyotrophic lateral sclerosis	Significant improvement	Significant improvement	21
Multiple sclerosis	Significant improvement	Symptoms alleviation	29
Spinal cord injury	Significant improvement	Significant improvement	36

## 3 Synergistic and Supplementary Roles of MSCs in the Therapy of Neurological Disorders

MSCs are still at a relatively early phase of clinical translation. Preclinical literature is abundant, while clinical trials are infrequent, and there is no MSC‐based product registered for neurological disorders. Therefore, the studies combining MSCs with other therapies are relatively rare, and if so, they are linked preferably with physical than pharmacological interventions. However, recently, Elbaz and co‐workers indicated that lercanidipine (LER) is neuroprotective in the stroke model, injected in the HD animal model before MSC transplantation, and augmented the MSC effect inhibiting neurological insults. Combined LER/MSCs therapy was revealed to be superior to MSCs alone, improving HD graft recipients' motor and behavioral abnormalities.^[^ ^94^, ^192^ ^]^ Accordingly, vitamin C augmented the therapeutic effects of MSCs in spinal cord injury.^[^ ^193^ ^]^ A traditional Chinese medicinal herb Icariin and MSCs synergistically promoted angiogenesis and neurogenesis after cerebral ischemia via Phosphoinositide 3‐kinases (PI3K) and Extracellular signal‐regulated kinases (ERK 1/2) pathways.^[^ ^194^ ^]^

Interestingly, the synergistic effect of treadmill exercise and transplanted MSCs was reported in the AD rat model. These studies showed that physical activity and MSC infusion increase MSC therapy's impact on memory impairment in AD rats.^[^ ^80^ ^]^ Treadmill exercise enhanced transplanted bone mesenchymal stem cells' therapeutic potency in cerebral ischemic rats via antiapoptotic effects.^[^ ^80^ ^]^ There were also observed synergic effects of rehabilitation and intravenous infusion of mesenchymal stem cells after stroke in rats.^[^ ^195^, ^196^ ^]^ The exposure of animals to an enriched environment enhanced angiogenesis after hypoxic–ischemic injury.^[^ ^197^ ^]^ There were also reported synergistic effects of mild hypothermia and adipose‐derived MSC for ischemic brain injury.^[^ ^198^ ^]^ There was also a synergy between MSC transplantation and repetitive transcranial magnetic stimulation on promoting autophagy and synaptic plasticity in vascular dementia.^[^ ^199^ ^]^ MSCs also synergize with electrical stimulation in Parkinson's disease.^[^ ^200^ ^]^

In summary, MSCs can act synergistically with other therapeutic strategies, and such a holistic approach is highly attractive, although costs might be limiting factors.

## 4 Methods of Transplantation Used in the Treatment of CNS Damage and Subsequent MSC Migration Pathways

The method of administration of transplanted stem cells has a significant impact on their location in the body, biodistribution within a given tissue, and the efficiency of their colonization of the affected area. In the case of damage to CNS structures, the most commonly used routes for cell administration include local and systemic transplantation and a relatively new nasal cell transplantation technique (**Table** **3**). Topical administration is usually performed by stereotactic injection of a cell suspension into the cerebrospinal fluid or directly into the brain parenchyma within the site of damage or in its proximity. This approach allows a huge number of transplanted cells to accumulate in a specific location; however, in the case of lesions of disseminated nature or covering a significant area of the organ, it does not ensure the proper distribution of transplanted cells. Also, a high accumulation of cells administered by topical injection may lead to premature death due to the limitation of efficient diffusion of oxygen and nutrients in such large clusters.^[^ ^201^ ^]^ A cell dosing device such as a cannula must be inserted into very sensitive brain tissues during the transplant procedure, causing bleeding or damage to the vital brain or spinal cord centers, leading to functional disorders in patients. After MSC intracerebral transplantation to a healthy brain, a low number of cells migrates from the injection site further into brain parenchyma. However, in transplantation to the corpus callosum, MSCs were observed to migrate along white matter tracts around cerebral blood vessels.^[^ ^202^ ^]^

**Table 3:** Characteristic of routes predominantly used for MSCs transplantation in CNS disorders

Administration route/characteristic	Intracerebral	Intrathecal	Intravenous	Intraarterial	Intranasal
Cells location in the body					
Directly after administration	Brain	Cerebrospinal fluid	Whole body circulation, Lungs, Liver, Spleen, Kidney microvessels	Brain	Brain
Secondary locations	Brain	Cerebrospinal fluid Perivascular Space Brain	Brain Liver Spleen Kidney	Brain Lungs Liver Spleen Kidney	Brain
Efficiency of affected brain area colonization	High	Low	Low	Medium	Unknown
Cells biodistribution within brain	Cumulated	Cumulated	Scattered	Scattered	Scattered
Invasiveness	High	High	Low	Medium	Low
Main risks	Brain hemorrhage Functional impairment	Backache Hydrocephalus	Pulmonary embolism	Micro‐embolisms	Inflammatory reactions Damage to the nasopharynx mucosa Allergic reactions

Intrathecal transplantation is the administration of cells by injection directly into the cerebrospinal fluid. In humans, the injection is usually made in the lower part of the spinal cord in the space between the arachnoid mater and pia mater. Intrathecally administrated cells may be transferred along with the circulation of cerebrospinal fluid to fluid spaces within the brain. Observation of intrathecally transplanted MSCs in a spinal cord injury model revealed their accumulation in the damaged area, with some cells penetrating to perivascular space in injured tissue.^[^ ^203^ ^]^ Kim et al. found that intrathecally injected MSCs spread to the brain area within 12 h from their administration while not present in other organs, including heart, lung, liver, spleen, and kidney. It was estimated that ≈2.4% of transplanted cells homed to the brain, thus a relatively low rate.^[^ ^204^ ^]^ In animals, after intrathecal injection, MSCs were detected in vivo by bioluminescence technique up to one week after transplantation.^[^ ^130^ ^]^ In another study, 24 h after injection, the genetic material of MSCs was detected in the spinal cord and heart of mice and hearts and brains of animals four months later.^[^ ^205^ ^]^ Both singular and repeated intrathecal injections of MSCs are currently being used in many clinical trials.^[^ ^140^, ^142^, ^206^ ^]^ Some patients complain of backache; however, the biggest problem connected with intrathecal cell delivery is the risk of abnormal cerebrospinal fluid flow, caused by cell‐induced obstruction, which can result in hydrocephalus.^[^ ^206^, ^207^ ^]^

Systemic transplantation is usually done by intravenous cell injection and characterized by a minimum surgical invasion level.^[^ ^91^, ^169^, ^208^ ^]^ In the case of intravenous administration, transplanted cells often appear in the damaged area of the brain a few days after injection in small numbers. By contrast, the arterial route seems to be an attractive combination of intracerebral and systemic administration advantages. Intra‐arterial administration of cells leads to their almost immediate delivery to the lesion region, usually in small clusters scattered throughout its space; also, it is performed without breaking the continuity of CNS structures, which is extremely sensitive to all manipulations.^[^ ^209^ ^]^ Thus, intra‐arterial administration can result in faster colonization of the lesion by more transplanted cells with a more homogeneous and extensive distribution area than intravenous injection. One of the basic problems associated with trans‐vascular transplantation of cells in the cell population's fate does not occupy the damaged area. According to the literature, up to 90% of intravenously injected cells accumulate in the pulmonary blood vessels.^[^ ^210^ ^]^ This can lead to pulmonary embolism, which is life‐threatening and reported in both animals and humans.^[^ ^211^, ^212^ ^]^ In some works, the authors address this by using remedies in the form of anticoagulants coadministration such as heparin, which seems to be a good approach this problem.^[^ ^213^, ^214^ ^]^ However, we should presume that its use will affect the adhesive capacity of the transplanted MSCs themselves and may affect the extent of colonization of the area of damage. Intravenous cells were also detected in other organs such as kidneys, spleen, and liver.^[^ ^215^ ^]^ In the intra‐arterial injection of cells, their deposition in the lungs is much smaller, and after injection, most of them are observed in the brain's damaged area.^[^ ^210^, ^216^ ^]^ MSCs intraarterially delivered to a healthy brain have been present in the motor and sensory cortex, hippocampus, striatum, thalamus, and hypothalamus.^[^ ^202^ ^]^ In most studies, the cells are observed inside brain blood vessels or close to at least three days after transplantation.^[^ ^202^, ^217^ ^]^ The time from brain damage to transplantation is important for the degree of CNS colonization by intraarterially delivered cells. The best results are achieved when the cells are transplanted within 48–72 h after injury. Administration of MSCs at earlier (24 h after injury) and later (7 days) time points leads to their negligible distribution within the lesion.^[^ ^218^ ^]^ The distribution and spreading of intra‐arterially injected cells are highly dependent on the hemodynamic state of brain vasculature. In the case of stroke, the second day after brain injury seems to be the most effective for cell transplantation with a relatively high homing rate due to lower edema and partial recanalization of blood vessels in the injured area directs the influx of cells. Nevertheless, a few hours after transplantation, many cells are moved to peripheral organs, mainly to the liver, kidneys, and spleen.^[^ ^219^ ^]^ Intra‐arterial transplantation may be associated with microembolism's risk due to the closure of the lumen of small‐diameter vessels in the brain by injected cells. Such situations have been observed simultaneously with a decrease in cerebral blood flow during infusion in animal models.^[^ ^220^ ^]^ A correlation was also found between the number of lesions following administration, the cell dose, and their administration velocity. However, our research results show that it is possible to avoid the appearance of unwanted side effects of intra‐arterial injections by precisely adjusting the infusion rate and the number of cells relative to their size.^[^ ^221^ ^]^

Intranasal administration was originally developed as a strategy for delivering drugs to CNS structures. Their penetration occurs through intracellular transport such as pinocytosis, endocytosis, or diffusion when drugs administered into the nasal cavity are absorbed by support cells, bipolar olfactory neurons, and the maxillary branch trigeminal nerve, which further delivers them to the olfactory bulb and other brain areas. It is also possible for the drug to penetrate the extracellular pathway, probably through connections between support cells or through spaces between them and olfactory cells. Substances supplied this way can penetrate the brain or cerebrospinal fluid. The administration's extracellular route provides a much shorter delivery time calculated in minutes, while the process takes from several hours to even several days for the intracellular pathway.^[^ ^222^ ^]^ In the case of some drugs, nasal administration is characterized by a much more efficient absorption of an active biological substance into the brain than an intravenous injection, which allows for a significant reduction in the administered agent's dose while maintaining the same level of effectiveness.^[^ ^223^ ^]^ Low‐molecular lipophilic compounds are delivered most effectively. Administration of cells via nasal route is difficult, mainly due to their significantly larger size.^[^ ^224^ ^]^ Although some studies call into question the effectiveness of cells delivered through the nasal pathway, other authors state that after transplantation of MSC in CNS diseases, they are observed in the area of the olfactory bulb, thalamus, hypothalamus, striatum, stump, and cortex.^[^ ^55^, ^225^, ^226^, ^227^ ^]^ Some MSCs were detected in the midbrain, striatum, and in the highest concentration in the olfactory bulb five days after intranasal administration; however, they were no longer visible after 7.5 weeks.^[^ ^103^ ^]^ Additional use of substances unsealing the nasopharyngeal mucosa, such as hyaluronidase, increases the area occupied by administrated cells visible in the brain after 1.5 h of their application.^[^ ^228^ ^]^ However, hyaluronidase application is associated with a risk of meningitis, allowing pneumococci and other bacteria to pass from the nasal cavity into the bloodstream.^[^ ^229^ ^]^ The simplicity of procedure conduction and its minimal invasiveness are undisputed advantages of intranasal cell administration. However, the nasal and systemic route's main disadvantage is the lack of control over the biodistribution of cells within the nervous system. Moreover, it should be mentioned that it is possible to experience adverse reactions in the form of inflammatory reactions, damage to the nasopharynx mucosa, and allergic reactions after intranasal cell application. Intranasal administration also avoids the risk of deposition of an excessive number of cells in peripheral organs. However, the mechanism of their penetration into the brain and the exact fate of the cells administered have not been explained well enough to be considered verified in terms of security and efficacy.

Systemic and intra‐arterial administration of cells is currently the most commonly used technique to treat CNS diseases using stem cell transplantation. One of the main problems associated with their use is that transplanted cells must leave the vascular bed to get to the brain affected by the disease. This process is hampered by the existence of significant BB‐specific barriers such as the BBB, blood‐cerebrospinal fluid barrier, and blood‐meninges barrier blood‐leptomeningeal barrier. Their role is to protect the CNS structures from the penetration of potentially harmful substances that may be present in the blood. In the physiological state, the existence of these barriers prevents cells' penetration into the brain parenchyma. Still, in response to damage, some of the immune system cells, such as leukocytes, acquire the ability to pass through the barriers mentioned above. This process is called diapedesis.

Molecular signaling pathways involved in MSC migration were predominantly investigated in vitro by analyzing cell transmigration through membrane pores of transwell chamber or wound healing – scratch assay. The majority of research investigated the chemoattractant effect, like a medium containing a high concentration of fetal bovine serum (FBS), on the migration rate of MSCs. A part of these studies selected growth factors that stimulate MSC migration, including transforming growth factor (TGF)‐*β* and fibroblast growth factor (FGF) families' members, platelet‐derived growth factor, Substance P, SDF‐1, VEGF‐C, proinflammatory cytokines such as Interferon (INF)‐*γ*, IL‐1*β*. It also has been shown that MSCs can stimulate their migration by autocrine signaling.^[^ ^230^, ^231^, ^232^, ^233^, ^234^, ^235^ ^]^ These molecules activate corresponding receptors on MSCs' surfaces. Interaction with TGF‐*β* occurs through the type 1 receptor, leading to the activation of both canonical and non‐canonical signal transduction pathways. VEGF‐C binds with VEGFR‐2 and VEGFR‐3 receptors, whereas SDF‐1 with chemokine receptor (CXCR)4 or CXCR7. INF‐*γ* provides Guanylate‐binding protein 1 (GBP1) receptor activation. Autocrine signaling stimulates the CXCR4 receptor and Aquaporin 1 (AQP1). Also, during migration, the activation of the Ca2+ permeable Piezo1 channel was detected.^[^ ^236^ ^]^ Intracellular kinases accomplish further signaling propagation. Some signaling pathways participating in MSC migration are commonly used by other cells for cytoskeleton remodeling during the cell division process. They begin from the activation of small Guanosine triphosphatases (GTPases) like Ras homolog gene family (Rho) and Cell division control protein 42 homolog (Cdc42), for which effector proteins constitute Rho‐associated protein kinase or mDia1. Other kinases with confirmed roles in MSC migration include PI3K‐Akt (phosphatidylinositol 3‐kinase/protein kinase B), ERK1/2 (extracellular signal‐regulated kinase 1/2), FAK (focal adhesion kinase), p38 MAPK (p38 mitogen‐activated protein kinase), Jak/Stat (Janus kinases/signal transducer and activator of transcription proteins), PYK2 (proline‐rich tyrosine kinase 2), MEK/ERK (mitogen‐activated protein kinase/extracellular signal‐regulated kinase), and ceramide kinase. Particular attention has been paid in the literature to the role of p38 MAPK kinase in MSC migration, the inhibition of which was shown to reduce movement of in vitro cultured MSCs significantly (72–87%).^[^ ^237^ ^]^ Activation of this signaling causes reorganization of cytoskeletal actin and myosin filaments and formation of stress fibers, thus creating focal adhesions and the cell's motile surface. This process is often accompanied by an increase in the expression of adhesion proteins and receptors like integrins *α*V*β*3, Lymphocyte function‐associated antigen 1 (LFA‐1), and CXCR4. Another type of MSC migration regulator is long noncoding (ribonucleic acids) RNAs. The terminal differentiation‐induced lncRNA (TINCR) was indicated as an inducer of cell movement toward chemoattractant by TINCR/ mir‐761/Wnt2 axis. TINCR is responsible for sponging, thus inactivating mir‐761, which regulates the Wnt2 expression. In MSC with TINCR overexpression, enhanced migration was observed concomitantly to high Wnt2 indication and elevation of *β*‐catenin and CXCR4 gene expression levels.^[^ ^238^ ^]^ An exciting aspect of MSC migration control is connected with post‐transcriptional protein changes. After MSCs exposition on bFGF, high‐mobility group 1A (HMGA1) and HMGA2 expression are elevated. It results in an increased level of enzymes responsible for core fucosylation (FUS8) and decreased proteins taking part in hydrolysis of core fucosylation like a‐l‐fucosidase 1 (FUCA1) and FUCA2. It provides the change of post‐transcriptional modifications of membrane‐associated proteins in MSC, including high sialylations and glycosylation. Since these post‐transcriptional changes are necessary for efficient ligands‐selecting binding, bFGF treatment possesses the potential to improve MSCs rolling on activated endothelium. With MSCs, one of the prominent adhesion protein families undergoing *N*‐glycosylation and fucosylation are integrins. The significance of post‐transcriptional protein modifications was revealed in a study where inhibition of FUS8 limited the dispersion of MSCs injected into zebrafish embryos and their migration to the injured bone in mice.^[^ ^239^ ^]^ Although previous in vitro studies have provided a lot of valuable information regarding the molecular basis of MSC migration mechanisms, they cannot fully explain the phenomena occurring in vivo.

**Figure 2:**
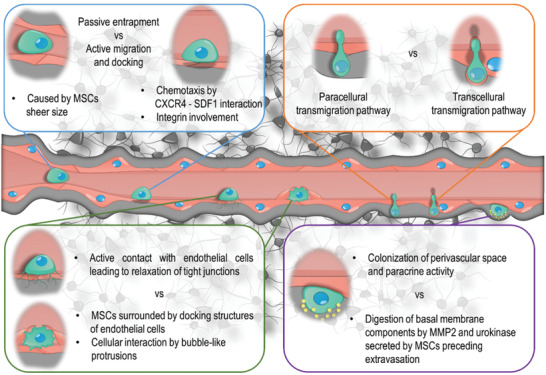
Comparing concepts available in the literature concerning each stage of MSC transmigration from blood vessel lumen to adjacent tissue.

MSCs are most often transplanted via the vascular system, which means transplanted cells must pass through the blood vessel wall to get into damaged structures. The process of migration and extravasation of MSCs is not yet well understood. Few papers describe its stages, and it seems that it is analogous to leukocyte diapedesis (**Figure** **2**).^[^ ^240^ ^]^ It is believed that the very first step of stopping MSCs administered systemically in the area of damage may be a passive phenomenon. It may result from the relatively large size of administered cells, which clog the lumen of small‐diameter blood vessels—such as capillary vessels.^[^ ^241^ ^]^ Nonetheless, receptors for chemotactic molecules such as CXCR4 (CXC 4 family chemokine receptor; CXC chemokine receptor type 4) are present on the surface of MSCs. There are also receptors from the CC subfamily chemokine binding group (CCR; chemokine receptor with CC motif; CC chemokine receptor), which can mediate the process of targeted migration and recruitment of systemically administered cells to the area of damage. The importance of CCR2 protein in the migration of intravenously administered MSCs to damaged myocardium and the role of CXCR4 receptor and its SDF‐1 ligand interactions have been documented thus far to direct the cell movement in the brains of animals after a hypoxic episode.^[^ ^242^, ^243^ ^]^ Unfortunately, apart from single examples, the contribution of the chemotaxis process to the particular organ of the systemically administered cells has not yet been adequately verified. When the MSCs get into the target area, where these cells will undergo transmigration, they stop. However, they do not have selectins on their surface, so their docking mechanism is different from that observed in leukocytes. MSCs are characterized by high expression of the CD44 protein—one of the significant selectin ligands, although due to the lack of fucosylation, it is not functional.^[^ ^244^, ^245^ ^]^ Therefore, assuming that the phenomenon of cell uptake from the bloodstream is an active process, the process of migration from the vascular bed to tissue must have a different molecular basis. Interactions of integrins with receptors from the immunoglobulin family are also indicated, which in leukocytes may be proteins involved in the alternative mechanism of cell rolling. Integrin subunits such as *β*1, *β*2, *α*1, *α*2, *α*3, *α*5, *α*6, and *α*V are present on the surface of MSCs. Reports in the literature about the expression of the *α*4 subunit offer conflicting data. Zuk, Orciani, and Pittenger state that MSCs do not express the *α*4 subunit. Other authors say that this protein is present and actively participates in the adhesion of MSCs to endothelial cells by interacting with the vascular cell adhesion molecule 1 (VCAM‐1) receptor.^[^ ^240^, ^246^, ^247^, ^248^, ^249^, ^250^ ^]^ Some studies have shown that the presence of *α*4 protein on MSCs may depend on the source of cell isolation. In the case of MSCs obtained from bone marrow, its expression level is insufficient to combine with VCAM‐1 functionally.^[^ ^244^ ^]^ The proportion of bone marrow MSCs expressing the *α*4 subunit, depending on published research results of various authors, ranges from 0.5% to 48%.^[^ ^240^, ^251^ ^]^ The reasons for the observed discrepancies may be related to different stages of the MSC cell cycle, their donor‐dependent heterogeneity, and differences in in vitro culture conditions, such as oxygen levels or cell density that have a massive impact on the expression profile adhesion proteins.^[^ ^252^, ^253^ ^]^ To date, it has been found that the *β*1 subunit plays an essential role in establishing contact between flowing MSCs and endothelial cells. The use of antibodies specifically blocking integrins or VCAM‐1 proteins present on endothelial cells reduces the number of cells retained and limits their migration to damaged myocardium in vivo. It indicates the involvement of these receptors in mechanisms controlling the uptake of MSCs from the bloodstream.^[^ ^254^, ^255^ ^]^ However, little is known about the importance of other integrins in initiating contact of MSCs with the endothelial layer. For leukocytes, binding receptors such as LFA‐1 and very late antigen‐4 (VLA‐4) to their ligands are keys in creating a stable connection with endothelial cells. Previous studies have shown that after the initiation of the MSCs’ contact with endothelial cells, polarization occurs within the MSCs’ cytoplasms. This phenomenon takes place due to the activation of the aforementioned CC chemokine receptors (CCR) receptor. The cytoplasmic end of this receptor is associated with an adapter protein called FROUNT, which triggers the PI3K phospholipase signaling cascade. It leads to the reorganization of the cytoskeleton and induction of protrusions production by stem cells. The above mechanism was first described in monocytes' chemotaxis and then identified in MSCs during their migration to the damaged myocardium.^[^ ^256^, ^257^ ^]^ MSCs have been shown to get in close contact with endothelial cells after binding to the endothelium layer, which leads to loss of tight junctions in the endothelial layer.^[^ ^258^ ^]^ In addition to endothelial transmission mechanisms described for leukocytes, such as para‐ and transcellular pathways, an alternative route called angiopellosis has been proposed for MSCs. Angiopellosis, unlike leukocyte diapedesis, is a process in which endothelial cells actively transfer passive MSCs from the light to the outside of the wall of the blood vessel. During this process, the transported cells retain their round shape.^[^ ^259^ ^]^ Furthermore, this phenomenon enables group transfer of cells.^[^ ^260^ ^]^ Based on the observation of the passage of MSCs through the endothelial cell layer in vitro, a model has also been proposed. MSCs do not migrate along the blood vessel but are surrounded by a docking structure and then produce vesicular projections in many directions. Because of these structures, MSCs encounter endothelial cells and cross their layer through unsealed tight junctions between them or pass through their cytoplasm. This process is similar to the extravasation of metastatic tumor cells and germ cells rather than leukocyte diapedesis.^[^ ^259^ ^]^ The passage of MSCs through the endothelium via a paracellular path appears to depend on PI3K kinase activity because its blocking inhibits cell transmigration; however, interactions between individual receptors during this process have not yet been described.^[^ ^261^ ^]^ A very interesting supposition was presented in the research work dedicated to the influence of MSCs’ nuclear envelope composition on transmigration. The researchers showed that MSC migration in vitro is lower than other mesodermal cell types. In the transwell migration assay, the initiation of MSC transmigration was observed. However, most cells stayed stacked on stage when large cytoplasmic parts of the cell body have already passed through the membrane pore, but the nucleus remained behind. Further studies revealed that MSC has irregularly organized nuclear envelope proteins like Lamin A/C, making the nucleus prone to wrinkling. Moreover, the ratio of Lamin A and Lamin B1 is atypical relatively to other mesodermal cells. These features of the nucleus might result in a lower successful transmigration rate of MSCs.^[^ ^262^ ^]^ Another obstacle on the path of cell transmigration is the basal membrane of the blood vessel, the crossing of which requires digestion of its components by proteolytic enzymes. In vitro studies show that MSCs can invade an artificial basement membrane model, and metalloproteinase 2 plays a key role in this process.^[^ ^263^ ^]^ Blocking expression of matrix metalloproteinase (MMP)2 has been shown to reduce the number of MSCs migrating through migration chambers coated with extracellular matrix proteins by up to 70%.^[^ ^264^ ^]^ According to some researchers, MSCs produce MMP‐2, while the second major leukocyte secretion metalloproteinase—MMP‐9 is not detected.^[^ ^250^ ^]^ Another proteolytic enzyme that appears to play an important role in the process of MSCs’ transmigration is urokinase, which has been identified in these cells' appendages during their passage through the endothelial layer in vitro.^[^ ^265^ ^]^ However, these proteases' importance during MSCs’ transmigration has not yet been confirmed in in vivo studies. In current literature, we found an increasing number of papers that showed that the perivascular space of blood vessels could be the destination site for transplanted MSCs within the damaged CNS area.^[^ ^202^, ^203^, ^217^ ^]^ Information can also be found about incorporating intravascularly administered MSCs into the vascular wall, probably through their integration into the endothelial cell layer.^[^ ^220^, ^250^, ^258^, ^266^ ^]^ According to the literature, the time needed for MSCs to migrate through endothelial cells is reported as 120 minutes (min), 240 min, 24 h, or 72 h.^[^ ^217^, ^240^, ^250^, ^258^, ^259^, ^267^ ^]^ One of the possible causes inducing the migration of cells from the lumen of the blood vessel to perivascular space may be developing hypoxia result in the death of a large proportion of cells due to low oxygen levels in clogged microvessels. This hypothesis seems likely in light of findings indicating the relationship between oxygen levels, integrin expression, and the ability of MSCs to migrate. Choi et al. showed that under hypoxia, HIF‐1 *α* translocates to MSCs nuclei and causes a decrease in Integrin Subunit Alpha 4 integrin expression, which in turn translates into enhanced MSC migration toward the chemotactic factor and an increase in MMP2 expression.^[^ ^252^ ^]^ This phenomena can lead to the initiation of MSCs’ migration process. Perivascular space is inhabited by pericytes with which MSCs share a similar surface antigen profile (CD146 +, CD34−, CD45−, CD56−) and the ability to differentiate into osteoblasts and chondrocytes. These and other consistent features of MSCs and pericites have led to the hypothesis of their common origin.^[^ ^268^, ^269^ ^]^ In the literature we can find works showing that in the native mouse and human brain within the perivascular space cells with MSCs features can be identified which indicates that perivascular space is the natural niche of MSCs in the brain.^[^ ^270^, ^271^ ^]^ From this location MSCs can actively exert neuroprotective and immunomodulatory effects and induce neurovascular unit regeneration through paracrine activity, extracellular vesicles release or filopodia extension.^[^ ^217^, ^272^, ^273^ ^]^

## 5 Techniques for Increasing the Colonization of the Lesion Area by MSCs Administered Systemically

In the light of current knowledge, stem cell transplantation is a promising direction for further developing regenerative medicine. However, the efficiency of migration and colonization of damaged organs by intravascularly administered MSCs is low, and the mechanisms controlling the above processes are not yet well understood. The increased influx and implantation of transplanted cells within affected organs may be a critical factor in cell therapy's success. To date, several types of strategies have been implemented to increase the migration of MSCs to target specific tissue and retain exogenously administered cells (**Table** **4**).

**Table 4:** Comparison of advantage, disadvantage, and bottleneck of techniques used for increasing the colonization of the lesion area by systemically administered MSCs

Method	Advantages	Disadvantages	Bottleneck
Pretreatment	Easy to perform No need for special equipment	No selective influence on specific cell characteristics The need for in‐depth phenotypical and functional analysis of cells after the procedure	The need for perfect repeatability of the procedure to ensure identical result Susceptible to multitude of environmental factors
Genetic modifications	Selective influence on the expression of a target gene Highly efficient	Low cell survival The risk of insertional mutagenesis Often requires special equipment The use of biological vehicles reduces safety of the procedures and hinders clinical translation	Difficult to maintain the balance between the efficiency of the procedure and the degree of cell damage Requires very solid optimization step Permanent overexpression of a given protein causing unpredictable, long‐term effects
Cell membrane engineering	Low time‐consuming High effectiveness Selective influence on cell properties	Temporal effect of modification	Scarce data from in vivo studies Difficult to select a single antigen to be modified given the complexity of the processes leading to cell homing
Changing the properties of the target tissue	Does not require the selection of a single molecule to drive cell homing Requires only a minimum level of cell modification (e.g., tagging)	Exposes a large area of the recipients’ body to field activity and changes induced thereby Tissue changes may persist long after transplantation and may cause side effects Requires specialized equipment	Maintenance of the balance between the Intensity of the applied field (increasing the inflow of cells) and the degree of tissue exposure to fields’ influence Necessity for careful verification of in vivo effect the field exerts on the tissue

### 5.1 Changes in In Vitro Culture Conditions (Pretreatment)

**Figure 3:**
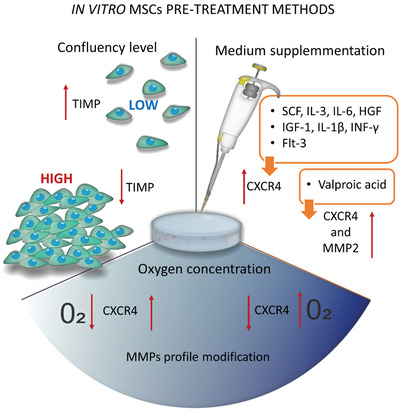
Aspects of in vitro culture conditions, which modification is indicated as a pretreatment method increasing MSC homing in vivo.

The standardization of in vitro cell culture protocols is designed to optimize conditions so that pretransplant cells have the properties of their native equivalents present in the body. Even small differences in ambient oxygen content, cell seed density, and culture medium composition can affect the MSC phenotype, including changes in the level of adhesive protein expression.^[^ ^253^ ^]^ Such a high sensitivity of cells to even slight differences in the environmental conditions was used to induce the expression of receptors and enzymes involved in cell migration and extravasation by supplementing the suspension in which they are maintained or modifying the culture conditions (**Figure** **3**). Due to the proven effect of CXCR4 on the targeted migration of MSCs in previous studies, stimulation of overexpression of this receptor was the target of in vitro manipulations.^[^ ^242^ ^]^ Induction or increase in CXCR4 expression can be obtained by supplementing the culture medium with a mixture of cytokines such as stem cell factor, IL‐3, IL‐6, HGF, and ligand flt ‐3 (tyrosine kinase 3 ligands) or with the addition of IGF‐1, IL‐1*β*, and IFN*γ*.^[^ ^274^, ^275^, ^276^ ^]^ Additionally, cell culture in the presence of valproic acid has been shown to enhance CXCR4 and MMP‐2 expression.^[^ ^277^ ^]^ Cell culture under hypoxia may be another experimental approach. Low oxygen concentration induces high expression of HIF‐1, which further results in elevated levels of SDF‐1 synthesis and CXCR4 expression.^[^ ^278^ ^]^ This process may be mediated by long noncoding RNA–LincRNA‐p21. LincRNA‐p21 stabilizes HIF‐1 in hypoxic conditions by limiting its binding with ubiquitin E3 ligase–VHL protein complex, which directs proteins on the degradation pathway.^[^ ^279^ ^]^ Studies show that the reduction of oxygen concentration increases CXCR4 synthesis and changes the profile of secreted metalloproteinases.^[^ ^280^, ^281^ ^]^ Obtaining the appropriate level of metalloproteinases and the number of receptors for chemokines is also possible by seeding cells at a specific density. Cells maintained in low confluence conditions secrete much smaller amounts of the tissue inhibitor of metalloproteinases (TIMP‐3) than densely growing cells and more efficiently invade the cell membrane model in vitro.^[^ ^263^ ^]^ MSCs overexpressing the CXCR4 receptor induced by a change in culture conditions show increased migration toward the SDF‐1 factor gradient, a specific ligand for CXCR4, and expanded accumulation lesion region in the model of enteritis, kidney, and bone marrow irradiation.^[^ ^263^, ^274^, ^275^, ^276^ ^]^ Similarly, a 60 min incubation of MSCs in a medium containing SDF‐1 protein increases the colonization of the damaged myocardial area by systemically administered cells.^[^ ^282^ ^]^ Another pretreatment approach used to manipulate the migration process is applying substances replacing animal serum in MSC culture medium. The platelet products trigger a more robust migration response of MSCs toward chemoattractant than standard FBS containing medium.^[^ ^283^ ^]^ According to in vitro observation, MSCs interact with platelets by podoplanin–transmembrane glycoprotein. This induces platelet aggregation, leading to creation of micro thrombi. Further, podoplanin rich plasmatic processes of MSCs were also shown to interact with endothelial cell's layer and cells with high expression of this glycoprotein possess enhanced migratory potential, at least in in vitro transwell assay.^[^ ^284^ ^]^ Changing the expression profile of gene coding proteins involved in the MSCs’ migration process by manipulating cell culture conditions in vitro is undoubtedly one of the technically easiest methods. Still, it is difficult to manage the process to obtain the effect desired by the researcher. The administered substances and changes in culture conditions have a much more multidirectional range of influence. It does not necessarily have a beneficial impact on the properties of the modified cells. Due to possible side effects, the use of specific experimental procedures requires a thorough analysis of the modified cells' phenotype and functionality.

### 5.2 Genetic Modifications of Cells

**Figure 4:**
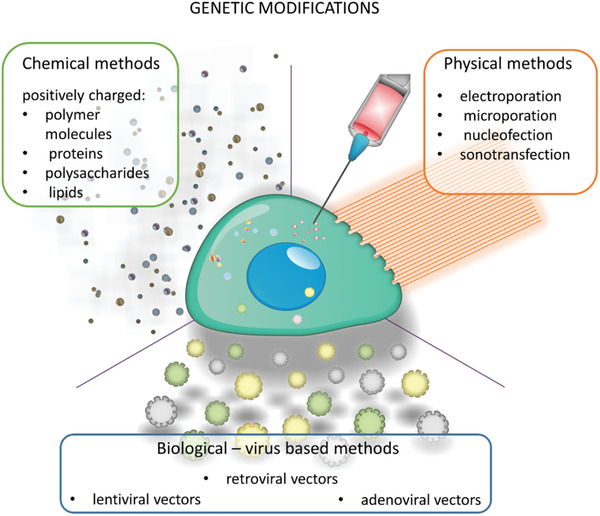
The most common methods used to obtain genetically modified MSCs.

One of the most commonly used techniques for changing cells' phenotype is to introduce into them expression vectors encoding a specific protein product (**Figure** **4**). Their carriers are very often viruses lacking the sequences responsible for reproduction and virulence. This employs the natural ability of viruses to invade eukaryotic cells and minimizes the risk of organ damage. The use of viral vectors to obtain transfection of MSCs is possible with relatively high but differentiated yields estimated at 35–100% of cells expressing the introduced construct.^[^ ^285^, ^286^ ^]^ Transduction with a retroviral vector containing the CXCR4 receptor coding sequence was used to obtain MSCs with increased potential for migration toward the SDF‐1 gradient, which allowed for increased colonization by such modified cells of bone marrow after their transplantation.^[^ ^287^ ^]^ Similarly, overexpression of the VLA‐4 receptor *α*4 subunit was successfully induced in MSCs after introducing an adenoviral carrier encoding this molecule, which increased the degree of colonization of mouse bone marrow.^[^ ^288^ ^]^ A lentiviral expression vector was used to induce CCR2 receptor overexpression in MSC; this increased their homing to stroke injured rat hemisphere after intravenous infusion.^[^ ^289^ ^]^ The stimulation of CX3C chemokine receptor 1 (CX3CR1) overexpression by lentiviral vector transduction provided enhanced accumulation of MSCs in the inflamed rat colon area, producing a high level of CX3CL1. Intravenously transplanted cells were shown to penetrate the extravascular space of the colon by transendothelial migration.^[^ ^290^ ^]^ The high efficiency of the discussed method is its undoubted advantage. At the same time, this technique carries the risk associated with the random placement of the viral carrier into the recipient's genetic material, which can initiate the phenomenon of insertional mutagenesis. Also, the host organism's immune response may be triggered by detecting the presence of virus particles.^[^ ^291^ ^]^ There are many alternatives to nonviral methods for introducing nucleic acid molecules into in vitro cultured cells, but they have lower yields. Plasmids and native DNA, miRNA, and mRNA molecules can be introduced into MSCs using various physical and chemical methods. Physical methods include electroporation, microporation, nucleofection, and sonotransfection. One example of using the physical transfection method in MSCs is the introduction of DNA molecules by electroporation to induce overexpression of the CXCR4 receptor. This process proceeded at 80% efficiency, enhancing the migration of transfected cells into the tumor‐occupied area after intracerebral administration into the contralateral hemisphere in the mouse glioma model.^[^ ^292^ ^]^ Similarly, the increase of MSC migration toward the SDF‐1 gradient was obtained due to nucleofection of mRNA encoding CXCR4, which was successfully performed in 93% of cells.^[^ ^293^ ^]^ However, it should be mentioned that all physical transfection techniques require disruption of the cell membrane of modified cells to allow transfection agent penetration, which is associated with damage and reduced viability.^[^ ^294^ ^]^ An alternative to physical techniques is those characterized by better cell survival and less efficient chemical methods, which include techniques based on the use of positively charged polymer molecules, proteins, polysaccharides, and lipids, which can bind to nucleic acids and help the cells’ internalization. The most commonly used method is called lipofection, which uses the possibility of nucleic acid encapsulation in liposomes. MSCs are described in the literature as cells resistant to chemical transfection techniques. Only about 2–35% of cells undergoing modification are obtained after introducing DNA molecules.^[^ ^295^ ^]^ Alternatively, it is possible to introduce mRNA into the cells, which allows for very rapid production of the protein product. The mRNA does not require translocation to the cell nucleus and transcription process; however, the expression of a specific protein obtained by this method is transient. Higher yields of transfection efficiency reaching 80–90% may be obtained. Such results were achieved, among others, using lipofection of MSCs’ mRNA encoding the CXCR4 receptor.^[^ ^296^ ^]^ Hitherto, the mRNA introduction was also used to induce *α*4 expression in MSCs, which enhanced cell adhesion in vitro and their initial settlement in the injured brain hemisphere after intra‐arterial injection.^[^ ^217^, ^297^ ^]^ It was shown that mRNA‐based transfection of MSCs with selectin ligands like P‐selectin glycoprotein ligand‐1 (PSGL‐1) and Sialyl‐Lewis is sufficient to increase the rolling of modified cells on activated endothelium surface in the microfluidic assay. It translated to the homing of engineered cells to EAE mouse spinal cord. Thus, it has been shown that it is possible to simultaneously introduce several exogenous mRNA transcripts to MSCs and obtain functional protein products.^[^ ^298^ ^]^ When it comes to clinical applications, a great advantage of the techniques based on the introduction of RNA molecules into cells is the lack of risk of an insertional mutagenesis process. They do not integrate into the genetic material of the recipient.^[^ ^293^ ^]^ However, so far, this technique has not widely been used to increase the colonization and migration capacity of MSCs to the in vivo injury region.

### 5.3 Cell Membrane Engineering

**Figure 5:**
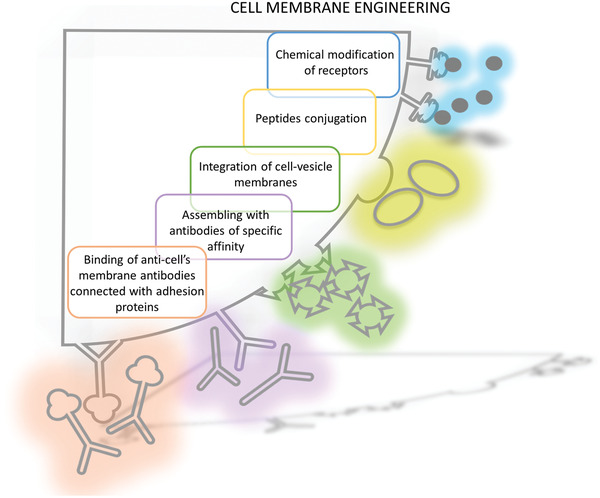
Strategies employed to modify MSCs cell membrane to increase targeted homing of cells.

Attachment of various molecules to the cell membrane or modification of the elements present on the surface of MSCs is a technique that has garnered particular interest in the last several years. There are several variants of this method based on a chemical reaction that modifies receptors present on the surface of cells or the attachment of whole proteins to the cell membrane.^[^ ^245^, ^299^ ^]^ It also is possible to introduce the protein of interest by forcing the integration of vesicles containing a specific receptor with the MSC's membrane or conjugation with an antibody connected to the appropriate receptor (**Figure** **5**).^[^ ^300^, ^301^ ^]^ To date, it has been attempted to have MSCs obtain the ability to bind to selectin family proteins using these techniques, which could support the first stage of their contact with activated endothelial cells. Pioneering work by Sackstein et al. involves a chemical reaction carried out leading to sialofucosylation of the inactive CD44 receptor present on the surface of MSCs, making it functional.^[^ ^245^ ^]^ E‐selectin binding protein K has also been chemically linked to the surface of MSCs.^[^ ^299^ ^]^ The SLEx domain (sialyl‐LewisX glycotope) of the PSGL‐1 receptor was instead attached to MSCs using an IgG antibody fragment and introduced into the MSC's cell membrane by integration with vesicles containing the structure on their cell membrane.^[^ ^300^, ^301^ ^]^ Induction of the presence of receptors for selectins on the surface of MSCs provided the cells with the ability to increase rolling, and adhesion in the fact of shear forces in blood vessel flow models in vitro as well as assisted colonization of modified bone marrow cells after systemic administration. Another exciting technique called “protein painting,” involves the attachment to the cell surface of antibodies with a specific affinity. In previous studies, anti‐intercellular adhesion molecule (ICAM)‐1 and VCAM‐1 proteins were added to MSCs, which increased the number of cells that bind to endothelial cells of blood vessels and increased colonization of the intestine by systemically administered cells in an inflammatory bowel disease model in mice.^[^ ^302^ ^]^ Studies in which the effect of MSC cell membrane engineering on these cells' properties did not reveal this procedure's harmful effects. The method's significant advantage is the relatively short time required to obtain cells with the desired properties.^[^ ^299^ ^]^ However, it should be kept in mind that the presence of the introduced modification obtained with this technique is usually temporary.^[^ ^300^ ^]^

### 5.4 Changing the Properties of the Target Tissue

**Figure 6:**
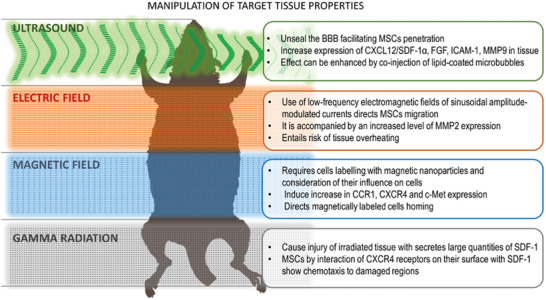
Techniques enhancing MSC homing by changing the destination area's properties under the influence of the external field.

Another method aimed at increasing the colonization of a given region by the system‐provided MSCs is to regulate this process by changing the properties of the target area itself (**Figure** **6**). An example is tissue irradiation, which causes increased influx and implantation of systemically administered cells in muscle, skin, intestine, and bone marrow exposed to gamma rays.^[^ ^303^, ^304^ ^]^ Similarly, the administration of substances/drugs used during radio‐ and chemotherapy that damage DNA leads to increased bone marrow colonization by transplanted MSCs, probably through the interaction of CXCR4 receptors present on cells with SDF‐1 secreted in large quantities by damaged tissue.^[^ ^305^ ^]^ It is also possible to control the migration of transplanted cells by the actions of an electric or magnetic field and an ultrasonic wave.^[^ ^306^, ^307^, ^308^ ^]^ The use of electric field has thus far been used only in in vitro studies due to the high risk of overheating the areas treated with it.^[^ ^306^ ^]^ However, MSCs placed in in vitro culture under the influence of low‐frequency electromagnetic fields of sinusoidal amplitude‐modulated currents retain their unchanged viability and concomitantly increased their migration toward chemoattractant in a transwell migration assay. It is accompanied by an increased level of MMP2 in MSCs. This effect seems to be specific for MSCs; thus, no other tested cell type (dendritic cells and fibroblasts) were influenced by electromagnetic field treatment.^[^ ^309^ ^]^ An attractive solution is to direct the migration of cells labeled with magnetic nanoparticles to a specific region by placing this area within the magnetic field. The strong magnetic properties of iron nanoparticles introduced into MSCs can increase the accumulation of labeled cells at the site of damage by applying an external source of the magnetic field in the lesion area. Placing an external magnet for 10 min in the injury region effectively enhanced the settlement of magnetically labeled MSCs injected directly into the extent of damage, shown in a patient with knee cartilage defect.^[^ ^310^ ^]^ This method has also been successfully used to obtain an accumulation of MSCs administered into the lesion area's damaged artery after angioplasty.^[^ ^311^ ^]^ Iron oxide nanoparticle labeling was shown to increase the number of migrating MSCs to chemoattractant in a transwell assay. At the molecular level, the labeling evoked an increase in CCR1, CXCR4, and c‐Met expression, all of these proteins are involved in migration control. Moreover, iron oxide labeling enhanced the migration to an inflammation site (ear infection) after intravenous injection of MSCs, even without external magnetic field application.^[^ ^312^ ^]^ However, not all nanoparticles exert a supportive role in migration stimulation of MSCs. Reduction in membrane fluidity and cytoskeletal abnormality were detected after MSCs labeled with magnetic nanoparticles containing a cobalt ferrite core coved by a silica shell. The disruption of migration rate in the chamber and limited activity in wound healing assay was observed. Thus, caution should be taken in establishing optimal labeling protocol and nanoparticles’ composition.^[^ ^313^ ^]^ The action of ultrasonic waves can be used to unseal the BBB, as demonstrated in experimental studies aimed at increasing drug delivery efficiency to CNS structures.^[^ ^314^ ^]^ This technique is known as focus ultrasound (FUS). FUS was shown to modulate genes in sonicated tissue in vivo, including cytokines, growth factors, adhesion molecules, and matrix remodelers (e.g., CXCL12/SDF‐1*α*, FGF, ICAM‐1, MMP9, respectively) and proved to affect the migration rate of MSCs at least in vitro. Thus this may encourage homing of these cells to FUS‐treated regions in vivo.^[^ ^315^ ^]^ In one study, sonification of the brain by FUS was shown to increase the expression level of ICAM‐1 and VCAM‐1 in tissue. ICAM‐1 elevated expression was observed mainly in glial cells and microglia, whereas VCAM‐1 expression was present especially in endothelial cells. MSCs transplanted systemically migrated more efficiently to sonificated brain probably due to more profound availability of adhesion protein in the tissue.^[^ ^316^ ^]^ The FUS strategy of BBB opening in some research is modified by coinjection of lipid‐coated microbubbles—a technique known as ultrasound‐targeted microbubble destruction. It seems that this method can also be used to facilitate the penetration of transplanted cells into areas of damage. Ultrasounds in even low diagnostic‐like intensity induces microbubble cavitation and bursting, facilitating a higher degree of local BBB disintegration. This is caused by enhanced shear and mechanical stress (known as shock wave) which affects endothelial cells membrane and may even induce permeabilization of blood vessel wall, enabling MSC penetration.^[^ ^317^ ^]^

## 6 In Vivo MSC Imaging

In vivo stem cell imaging is essential for both their precise deployment and understanding of their therapeutic activity. There are various approaches to MSC tagging and imaging, depending on their experimental and clinical needs. Superparamagnetic iron oxide nanoparticles (SPIONs) are quite versatile and relatively frequently used cellular label. It has been shown that SPIONs are mostly neutral to MSCs in vitro^[^ ^318^ ^]^ and in vivo.^[^ ^319^ ^]^ A very high signal of SPIONs, which allows them to be detected within seconds, is a major advantage. This property facilitates real‐time imaging of their delivery to the brain.^[^ ^218^ ^]^ There were also not found negative consequences of labeling by SPIONs on the therapeutic potential of MSCs.^[^ ^320^, ^321^ ^]^ Some studies even claim the single‐cell detection of iron oxide labeled MSCs transplanted to the rat brain in MRI.^[^ ^322^ ^]^ However, the major disadvantage of SPIONs is an inherently negative signal in MRI, which precludes a high specificity of a signal. SPIONs are permanently kept in the tissue, thus inept at reporting on cell survival.^[^ ^323^ ^]^ Fluorine nanoemulsion is another cellular tag. It is characterized by low sensitivity and the need for advanced MRI equipment, making the whole process more complicated. Luckily, fluorine nanoemulsion is also not toxic to MSCs in vitro^[^ ^324^ ^]^ and in vivo.^[^ ^325^ ^]^ The particularly desirable property of fluorine nanoemulsion is its disappearance upon cell death, so it might be used to report on cell survival. However, any direct labeling methods are not applicable for long‐term studies as the tags are diluted over the proliferation of transplanted cells.

Reporter genes are the holy grail of cellular imaging, though they are entirely incompatible with MSC workflow. MSCs are typically derived from various tissues, and after short expansion, they are transplanted as a primary cell population. Therefore, they are particularly prone to the adverse impact of genetic manipulations. Despite that, single studies present no genetic engineering implications with magnetic reporter gene on MSCs properties.^[^ ^326^ ^]^ The limited sensitivity of magnetic reporter genes makes them mostly obsolete. However, single studies demonstrate their utility for MSC tracking in the brain.^[^ ^327^ ^]^ Thus the application of radioactivity in conjunction with reporter genes is a practical approach. However, the minimal BBB penetration of radioisotopes makes the brain a particularly challenging target.^[^ ^328^ ^]^ The bioluminescent and optoacoustic imaging are very robust in small animal settings, while they are not useful for large animal and clinical studies.^[^ ^329^ ^]^ Reporter genes also have a potential for an insight into a differentiation or transdifferentiation of transplanted MSCs.^[^ ^330^ ^]^

Overall, direct labels and reporter genes are two classes of tagging strategies with their strengths and drawbacks. The selection of an MSC imaging method must be meticulous and tailored to the specific scientific inquiries.

## 7 Summary and Conclusions

In summary, experimental studies using animal models and the first clinical trials of MSC transplantation in CNS diseases indicate that these cells have positive therapeutic effects. Their transplantation is safe and does not appear to cause undesirable side effects. The available literature shows that MSCs are actively mobilized to damaged tissues, but the mechanism responsible for this process has not yet been discovered. The colonization of tissue damage regions by systemically administered cells is much less efficient in MSCs than in leukocytes, which may be caused by the lack of expression of receptors for chemokines and adhesion proteins important for this process. An incredibly difficult task is to induce the colonization of CNS structures by transplanted cells due to the BBB. Therefore, the development of methods to increase the targeted migration of systemically administered cells to the brain is essential to increase the efficiency of therapies using exogenous MSCs in neurodegenerative diseases. The thorough summary of data extracted from individual papers is present in Table S1 of the Supporting Information.

## Conflict of Interest

The authors declare no conflict of interest.

## Supporting information

Supporting Table

Supporting Table

Supporting Information
