# Supplementary material for: Mesenchymal Stem Cells for Neurological Disorders
Source: Adv Sci (Weinh). 2021 Feb 24;8(7):2002944. doi: 10.1002/advs.202002944 (PMC8024997; doi:10.1002/advs.202002944)
Supplement: Supplementary file 3 — Supporting Information [file ADVS-8-2002944-s003.pdf]

© 2020 Wiley-VCH GmbH

ADVANCED SCIENCE
Open Access

Supporting Information

for *Adv. Sci.,* DOI: 10.1002/advs.202002944

Mesenchymal Stem Cells for Neurological Disorders

*Anna Andrzejewska, Sylwia Dabrowska, Barbara Lukomska, and Miroslaw Janowski**
